# Analysis of Sociodemographic, Clinical, and Genomic Factors Associated With Breast Cancer Mortality in the Linked Surveillance, Epidemiology, and End Results and Medicare Database

**DOI:** 10.1001/jamanetworkopen.2021.31020

**Published:** 2021-10-29

**Authors:** Timothy J. Robinson, Lauren E. Wilson, P. Kelly Marcom, Melissa Troester, Charles F. Lynch, Brenda Y. Hernandez, Edgardo Parrilla, Heather Ann Brauer, Michaela A. Dinan

**Affiliations:** 1 Department of Radiation Oncology, H. Lee Moffitt Cancer Center, Tampa, Florida; 2 Department of Biostatistics and Bioinformatics, H. Lee Moffitt Cancer Center, Tampa, Florida; 3 Department of Population Health Sciences, Duke University School of Medicine, Durham, North Carolina; 4 Department of Medical Oncology, Duke Cancer Institute, Duke University, Durham, North Carolina; 5 Lineberger Comprehensive Cancer Center, University of North Carolina at Chapel Hill; 6 Department of Epidemiology, Gillings School of Global Public Health, University of North Carolina at Chapel Hill; 7 College of Public Health, University of Iowa, Iowa City; 8 University of Hawaii Cancer Center, University of Hawaii at Mānoa, Honolulu; 9 Department of Pathology, Duke University School of Medicine, Durham, North Carolina; 10 Discovery and Translational Sciences, Bill and Melinda Gates Foundation, Seattle, Washington; 11 Department of Chronic Disease Epidemiology, Yale School of Public Health, New Haven, Connecticut; 12 Yale Cancer Outcomes, Public Policy, and Effectiveness Research Center, New Haven, Connecticut

## Abstract

**Importance:**

Understanding interactions among health service, sociodemographic, clinical, and genomic factors in breast cancer disparities research has been limited by a disconnect between health services and basic biological approaches.

**Objective:**

To describe the first linkage of Surveillance, Epidemiology, and End Results (SEER)–Medicare data to physical tumor samples and to investigate the interaction among screening detection, socioeconomic status, tumor stage, tumor biology, and breast cancer outcomes within a single context.

**Design, Setting, and Participants:**

This population-based cohort study used tumor specimen blocks from a subset of women aged 66 to 75 years with newly diagnosed nonmetastatic, estrogen receptor–positive invasive breast cancer from January 1, 1993, to December 31, 2007. Specimens were obtained from the Iowa and Hawaii SEER Residual Tissue Repositories (RTRs) and linked with Medicare claims data and survival assessed through December 31, 2015. Data were analyzed from August 1, 2018, to July 25, 2021.

**Exposures:**

Screening- vs symptom-based detection of tumors was assessed using validated claims-based algorithms. Demographic factors and zip code–based educational attainment and poverty socioeconomic characteristics were obtained via SEER.

**Main Outcomes and Measures:**

Molecular subtyping and exploratory genomic analyses were completed using the NanoString Breast Cancer 360 gene expression panel containing the 50-gene signature classifier. Factors associated with overall and breast cancer–specific (BCS) survival were analyzed using Cox proportional hazards regression models combining sociodemographic, clinical, and genomic data.

**Results:**

SEER-Medicare data were available for 3522 women (mean [SD] age, 70.9 [2.6] years; 3049 [86.6%] White), of whom 1555 (44.2%) were diagnosed by screening mammogram. In the SEER-Medicare cohort, factors associated with increased BCS mortality included symptomatic detection (hazard ratio [HR], 1.49 [95% CI, 1.16-1.91]), advanced disease stage (HR for stage III, 2.33 [95% CI, 1.41-3.85]), and high-grade disease (HR, 1.85 [95% CI, 1.46-2.34]). The molecular cohort of 130 cases with luminal A/B cancer further revealed increased all-cause mortality associated with genomic upregulation of transforming growth factor β activation and p53 dysregulation (eg, p53 dysregulation: HR, 2.15 [95% CI, 1.20-3.86]) and decreased mortality associated with androgen receptor, macrophage, cytotoxicity, and Treg signaling (eg, androgen receptor signaling: HR, 0.23 [95% CI, 0.12-0.45]). Symptomatic detection (HR, 2.49 [95% CI, 1.19-5.20]) and zip codes with low levels of educational attainment (HR, 5.17 [95% CI, 2.12-12.60]) remained associated with mortality after adjusting for all clinical and demographic factors.

**Conclusions and Relevance:**

Linkage of SEER-Medicare data to physical tumor specimens may elucidate associations among biology, health care access, and disparities in breast cancer outcomes. The findings of this study suggest that screening detection and socioeconomic status are associated with survival in patients with locally advanced, estrogen receptor–positive tumors, even after incorporating clinical and genomic factors.

## Key Points

**Question:**

Can existing data sets be used to link sociodemographic, clinical, and genomic data into a single population-level data set to investigate disparities in cancer outcomes?

**Findings:**

This cohort study used first-in-kind linkage of Surveillance, Epidemiology, and End Results, Medicare, and residual tumor repository data of 3522 women with newly diagnosed screening- vs symptomatic-detected estrogen receptor–positive nonmetastatic breast cancer to demonstrate that screening and socioeconomic factors remain associated with breast cancer outcomes, even after adjusting for clinical, demographic, and genomic factors.

**Meaning:**

These findings suggest that screening detection, tumor stage, gene expression, and survival are associated phenomena that may offer novel insights when examined together within a single context.

## Teaser

This cohort study uses linked registry data to investigate the biological and clinical progression of cancer associated with sociodemographic data and screening vs symptomatic detection in women with nonmetastatic invasive estrogen receptor–positive breast cancers.

## Introduction

Despite advances in our basic understanding of breast cancer biology, the relative contribution of sociocultural and biological factors in breast cancer disparities has remained an area of active debate during the past 30 years, and pure biological, social, and care access–based models cannot accurately describe all epidemiological phenomena.^1,2^ Evidence of social drivers of race-based disparities has been demonstrated with respect to screening, stage at detection, treatment, and overall survival.^3,4,5,6^ Poverty is associated with advanced-stage disease presentation,^7^ and increased distance to care is associated with decreased use of adjuvant therapy.^8^ On the other hand, analyses of phase III SWOG trials have demonstrated disparities in breast cancer outcomes, even in the setting of presumably equal care.^9^ Furthermore, many features of breast cancer, including receptor status, remain stable during the course of metastatic cancer, suggesting that these molecular subtypes reflect different biological entities,^10,11,12^ and genomic risk scores have prognostic and predictive capability 10 years after initial treatment.^13,14,15,16^

To better understand breast cancer disparities, investigations of “nature and nurture”^17^ must be combined, accounting for population sciences and dissemination of cancer care.^18^ Most breast cancer research addresses basic science, health services, or clinical domains, but rarely all 3. A key driver of this siloed research is the paucity of population-level linkage containing both clinical and health services data with physical tumor samples. Last, most genomically analyzed tumor samples are collected in academic medical centers or within the context of a clinical trial, settings known to differ substantially from the general population with respect to patients, treatment, and outcomes.^19,20,21,22^

In this study, we conducted a proof-of-principle transdisciplinary investigation of health services and basic biological data within a population-level sample of patients. To accomplish this, we linked Surveillance, Epidemiology, and End Results (SEER) data, physical tumor blocks from the SEER residual tumor repository (RTR), and associated Medicare claims data. We then used this novel data set to investigate the biological and clinical progression of cancer associated with sociodemographic data and screening vs symptom detection among women with nonmetastatic invasive estrogen receptor (ER)–positive breast cancers in a population-level study. The primary aim of our study was to demonstrate the feasibility of our approach to investigate the interaction among health service, demographic, and clinical factors and their association with breast cancer–specific (BCS) and overall survival after adjusting for genomic factors. Our secondary aim was to investigate the association among health service, demographic, and clinical factors with tumor biology and progression.

## Methods

### Data Source

This cohort study was approved by all participating entities’ individual institutional review boards, which waived the need for informed consent owing to the use of deidentified registry data. This study followed the Strengthening the Reporting of Observational Studies in Epidemiology (STROBE) reporting guideline.

The RTR banks formalin-fixed, paraffin-embedded (FFPE) blocks of tumor tissues that were clinically discarded, including primary, lymph node, and metastatic tumors from patients diagnosed in Iowa and Hawaii from January 1, 1993, to December 31, 2007. SEER data are linked with these physical tumor blocks, providing basic clinical and demographic information (eg, age, race, stage). SEER-coded race and ethnicity are determined per the SEER coding manual, which is primarily based on self-reported race and ethnicity as contained within the electronic medical record. Medicare insures approximately 97% of Americans 65 years or older, and administrative claims data are collected as part of routine operation, with deidentified claims data serving as a commonly used research data set. These data include all Medicare-billed services received by a patient, and therefore provide detailed and accurate data regarding the longitudinal treatment of patients. Linked Medicare claims data from January 1, 1992, through December 31, 2008, were available for analysis. This linkage represents, to our knowledge, the first joint data set combining the SEER, SEER-RTR, and Medicare claims data.

### Study Population and Analysis Cohorts

**Figure 1.**
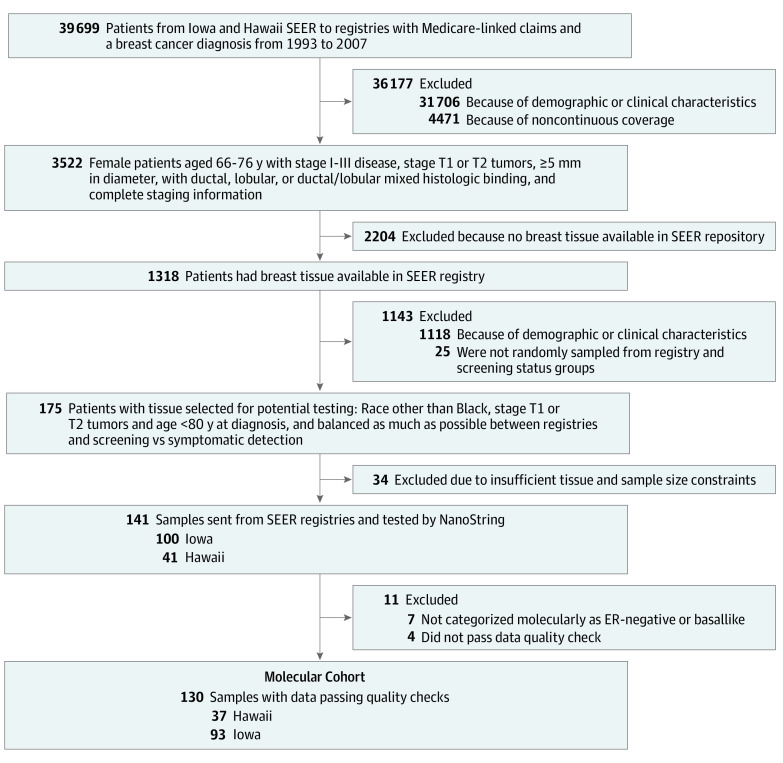
Study Cohort Composition and Inclusion and Exclusion Criteria A Surveillance, Epidemiology, and End Results (SEER)–Medicare cohort was created from women who met clinical inclusion and exclusion criteria and were diagnosed from 1993 to 2007 within the Iowa and Hawaii SEER catchment regions. A total of 3522 women met further clinical and claims criteria (see Methods) and constituted the SEER-Medicare cohort. This cohort was used to conduct a standard health services investigation, including confirmation of the association between our metric of screening and outcomes. Of the 3522 patients within the SEER-Medicare cohort, 1318 (37.4%) had available formalin-fixed, paraffin-embedded blocks. A subset of these patients was selected for genomic analysis, stratifying by screening status to create the final molecular cohort of 130 individuals. ER indicates estrogen receptor.

A SEER-Medicare cohort was created using all patients who met study criteria and for whom both SEER and Medicare claims data were available (eFigure 1 in the Supplement) and included women with a SEER-based diagnosis of ER-positive invasive breast cancer from 1993 to 2007 with a confirmatory inpatient, outpatient, or carrier-based Medicare claim. Standard SEER-Medicare inclusion and exclusion criteria were then applied (Figure 1). We excluded T3 and T4 tumors because these would likely have only been symptomatic. Patients were required to be 66 years or older per standard SEER-Medicare study inclusion criteria. We limited our study to women 75 years or younger to focus on women who were more likely to undergo treatment with reasonable remaining natural life expectancy, and we included women with prior malignant disease. A subset of the SEER-Medicare cohort was then used to create a molecular cohort. Cases were selected by evenly sampling from screening- vs non–screening-detected tumors for which tissue samples were available, limited to those with adequate RNA integrity for genomic analysis, and further limited to samples confirmed as either luminal A or B cancer by molecular subtyping with a 50-gene signature (PAM50). Central pathological confirmation of all tumor cases and grade determination was performed by a single breast cancer pathologist (E.P.).

### Primary Study End Points

Screening detection of tumors was determined using the presence of a bilateral screening mammography in the 4 months before the breast cancer diagnosis claim or in the year before the site-directed breast surgery as validated previously.^23,24^ The National Cancer Institute comorbidity index was determined using inpatient, outpatient, and carrier claim files in the year before diagnosis.^25,26^ For SEER stage, we used the American Joint Committee on Cancer Staging Manual, third edition, from 1993 to 2003 and the sixth edition for tumors diagnosed after 2003. Patient demographics included 2003 rural/urban continuum codes and zip code–based neighborhood socioeconomic status. Patient mortality and cause-of-death data were sourced from the SEER-Medicare data set, which included mortality follow-up through December 31, 2015. Study follow-up was censored at the point where 25% of the initial cohort remained, which was at 15.1 years after breast cancer diagnosis.

### Genomic Analyses

The FFPE tumor specimens were analyzed using a gene expression profiling (Breast Cancer 360 [BC360]; NanoString, Inc) to quantify continuous values for the messenger RNA expression of 752 genes and 30 cancer-related gene expression signatures (eg, androgen receptor signaling) and provide molecular subtyping into luminal A, luminal B, *ERBB2* (formerly known as *HER2*)–enriched, and basallike using 58 genes and the PAM50 algorithm. Heatmaps of expression profiles were created using hierarchical clustering with nSolver, version 4.0 (NanoString) and the R heatmap statistical package (R Program for Statistical Computing) for exploratory analyses of gene signature clustering in screen- and symptom-detected cancer as well as by T and N stage. Expressions of 752 genes were individually regressed as continuous values as a function of screening status, T stage, N stage, and association with BCS and overall survival controlling for clinical, demographic, and socioeconomic factors using a threshold of unadjusted *P* < .05 for exploratory analyses. Where reported, false discovery rate was calculated using the Benjamini-Hochberg correction. The total number of samples obtained was restricted by project resources, which allowed the molecular analysis of 140 samples.

### Statistical Analysis

Data were analyzed from August 1, 2018, to July 25, 2021. Associations between screening status and stage were analyzed using bivariate *t* tests and the Cochran-Mantel-Haenszel test for nonzero correlation as well as unadjusted and adjusted logistic regression. Survival analyses were performed using unadjusted and adjusted Cox proportional hazards regression to estimate the associations between both BCS and all-cause mortality and patient demographic and socioeconomic factors, stage at diagnosis, screening detection status, and tumor molecular characteristics, including molecular subtype and ER/progesterone receptor expression status. Variable collinearity was assessed using the variance inflation factor, and variables with a variance inflation factor of greater than 10 and with a lower *R*^2^ value were removed for the final model. Significance testing was performed using 2-sided tests with α = .05. Statistical analyses were performed using SAS version 9.4 (SAS Institute Inc) and RStudio version 1.2.1139 (RStudio Inc).

## Results

### SEER-Medicare Cohort: Association Between Patient Characteristics, Stage, and Outcomes

Within the cohort of patients with SEER-Medicare linked data, a total of 3522 women (mean [SD] age, 70.9 [2.6] years) met inclusion criteria; of these patients, 598 (17.0%) were from the Hawaii SEER registry and 2924 (83.0%) were from the Iowa SEER registry. Patients were limited to 66 to 75 years of age by inclusion criteria, with 1557 (44.2%) aged 66 to 70 years. The cohort included 338 East Asian patients (9.6%), 72 Native Hawaiian patients (2.0%), and 3049 White patients (86.6%), with Black patients and those of unknown or other race constituting numbers too small to report per standard SEER-Medicare data use agreements limiting reporting of cell sizes of fewer than 11. A total of 1555 patients (44.2%) had screen-detected and 1967 (55.8%) had symptom-detected tumors. Screening detection was more common among patients with T1 (1316 of 2630 [50.0%]) vs T2 (239 of 892 [26.8%]) tumors. Patients with T3 tumors (23 of 163 [14.1%] screen detected) were excluded from the study. No association was observed between screening detection and whether a patient’s breast cancer diagnosis was their first-ever recorded cancer, which was the case for 3206 patients (91.0%). In multivariable analysis, symptom-detected tumors were associated with higher T stage (odds ratio [OR] for T2 vs T1, 2.70 [95% CI, 2.27-3.21]) and more advanced nodal involvement (OR for N2-N3 vs N1-N0, 1.79 [95% CI, 1.31-2.43]) (Table 1). The only other factors associated with more advanced T stage or N stage disease were high-grade tumors (ORs, 2.06 [95% CI, 1.72-2.46] and 1.54 [95% CI, 1.12-2.11], respectively) and lobular histologic findings (ORs, 1.85 [95% CI, 1.43-2.39] and 1.95 [95% CI, 1.29-2.95], respectively). Symptom-detected disease was further associated with all-cause mortality (hazard ratio [HR], 1.21 [95% CI, 1.09-1.35]) and BCS mortality (HR, 1.49 [95% CI, 1.16-1.91]). In addition to symptomatic detection, factors associated with increased BCS mortality included disease stage (HR for stages I vs II, 0.27 [95% CI, 0.21-0.34]; HR for stages III vs II, 2.33 [95% CI, 1.41-3.85]) and high-grade disease (HR, 1.85 [95% CI, 1.46-2.34]). All-cause mortality was also associated with more advanced stage (HR for stages III vs II, 1.44 [95% CI, 1.02-2.04), higher-grade tumors (HR, 1.29 [95% CI, 1.15-1.45]), older age (HR for 71-75 vs 66-70 years, 1.37 [95% CI, 1.24-1.52]), comorbidities (HR for 1, 1.78 [95% CI, 1.60-1.99]; HR for ≥2, 3.11 [95% CI, 2.70-3.58]), and being unmarried (HR, 1.20 [95% CI, 1.09-1.33]). Native Hawaiian race was associated with increased all-cause mortality (HR, 1.55 [95% CI, 1.17-2.06]), whereas East Asian race was associated with decreased all-cause mortality (HR, 0.55 [95% CI, 0.45-0.67]), but there were no racial differences in cancer-specific mortality (HRs, 0.41 [95% CI, 0.13-1.30] and 0.76 [95% CI, 0.49-1.18], respectively).

**Table 1.** Surveillance, Epidemiology, and End Results–Medicare Cohort: Multivariable-Adjusted Analysis of Factors Associated With Higher T Stage, N Stage, All-Cause Mortality, and BCS Mortality (N = 3522)

	OR (95% CI)		HR (95% CI)	
	T stage (T2 vs T1)	N stage (N2-N3 vs N1-N0)	All-cause mortality	BCS mortality
Symptomatic detection	2.70 (2.27-3.21)	1.79 (1.31-2.43)	1.21 (1.09-1.35)	1.49 (1.16-1.91)
Stage at diagnosis				
I	NA	NA	0.66 (0.59-0.73)	0.27 (0.21-0.34)
II	NA	NA	1 [Reference]	1 [Reference]
III	NA	NA	1.44 (1.02-2.04)	2.33 (1.41-3.85)
Tumor grade				
I/II	1 [Reference]	1 [Reference]	1 [Reference]	1 [Reference]
High (III)	2.06 (1.72-2.46)	1.54 (1.12-2.11)	1.29 (1.15-1.45)	1.85 (1.46-2.34)
Missing	1.18 (0.88-1.59)	1.13 (0.69-1.85)	1.16 (0.98-1.39)	1.07 (0.71-1.63)
Age at diagnosis, y				
65-70	1 [Reference]	1 [Reference]	1 [Reference]	1 [Reference]
71-75	0.99 (0.85-1.17)	1.18 (0.89-1.57)	1.37 (1.24-1.52)	1.12 (0.90-1.40)
Race and ethnicity				
Black	1.16 (0.33-4.06)	2.97 (0.62-14.3)	0.86 (0.38-1.94)	2.64 (0.96-7.26)
East Asian	0.79 (0.58-1.07)	1.43 (0.87-2.35)	0.55 (0.45-0.67)	0.76 (0.49-1.18)
Native Hawaiian	1.43 (0.85-2.40)	2.00 (0.88-4.53)	1.55 (1.17-2.06)	0.41 (0.13-1.30)
White	1 [Reference]	1 [Reference]	1 [Reference]	1 [Reference]
Other^a^	0.95 (0.49-1.84)	1.61 (0.56-4.64)	0.53 (0.34-0.84)	0.20 (0.03-1.45)
Comorbidity score				
0	1 [Reference]	1 [Reference]	1 [Reference]	1 [Reference]
1	1.05 (0.87-1.26)	0.95 (0.68-1.33)	1.78 (1.60-1.99)	1.11 (0.85-1.44)
≥2	1.09 (0.84-1.42)	0.79 (0.48-1.31)	3.11 (2.70 − 3.58)	0.89 (0.57-1.40)
Zip code at diagnosis (top quartile)				
Black race	0.91 (0.74-1.11)	1.01 (0.70-1.45)	1.00 (0.88-1.13)	1.06 (0.80-1.39)
Did not finish high school	0.85 (0.70-1.03)	1.08 (0.78-1.51)	1.01 (0.90-1.13)	1.26 (0.98-1.63)
Low-income household	1.20 (1.00-1.46)	0.98 (0.70-1.38)	1.05 (0.93-1.18)	1.04 (0.80-1.36)
Married	0.90 (0.76-1.06)	0.93 (0.70-1.24)	0.83 (0.75-0.91)	0.96 (0.77-1.20)
Lives in metropolitan area	1.01 (0.85-1.21)	0.77 (0.55-1.06)	0.99 (0.88-1.10)	1.08 (0.84-1.38)
Lives in rural area	0.90 (0.65-1.24)	1.18 (0.71-1.99)	0.92 (0.75-1.12)	0.93 (0.60-1.45)
Histologic finding				
Ductal	1 [Reference]	1 [Reference]	1 [Reference]	1 [Reference]
Ductal/other	1.18 (0.55-2.50)	1.00 (0.23-4.25)	1.03 (0.67-1.58)	0.34 (0.05-2.43)
Lobular	1.85 (1.43-2.39)	1.95 (1.29-2.95)	0.90 (0.76-1.07)	1.24 (0.88-1.74)
Lobular/ductal	1.35 (0.99-1.84)	1.46 (0.87-2.45)	0.92 (0.75-1.12)	1.09 (0.71-1.67)
Progesterone receptor status				
Positive	1 [Reference]	1 [Reference]	1 [Reference]	1 [Reference]
Borderline/negative	1.18 (0.96-1.45)	1.29 (0.91-1.81)	1.10 (0.97-1.24)	1.25 (0.96-1.63)
Missing	0.50 (0.19-1.33)	0.46 (0.06-3.40)	0.62 (0.34-1.12)	NA
Diagnosed in 2000 or later	1.10 (0.92-1.31)	0.94 (0.69-1.28)	1.01 (0.91-1.12)	0.79 (0.62-1.02)

Abbreviations: BCS, breast cancer–specific; HR, hazard ratio; NA, not applicable; OR, odds ratio.

^a^ Includes American Indian/Alaska Native, other Asian/Pacific Islander, South Asian, and other/not specified.

We next investigated factors associated with screening detection. In univariate comparisons we found that screen-detected tumors were smaller and more often T1 vs T2 (1315 of 1555 [84.6%]) than symptomatic tumors (1314 of 1967 [66.8%]) and that patients with symptomatic tumors were more likely to reside in the zip code with the lowest quartile of high school graduation rates than patients with screen-detected tumors (567 of 1967 [28.8%] vs 321 of 1555 [20.6%]) (eTable 1 in the Supplement). The proportion of tumors that were screen detected increased during the study period, with persistently higher rates in 2000 or later (eFigure 2 in the Supplement). In multivariable analyses, we found that patients with symptomatic disease were more likely to have higher stage disease (OR for stages III vs II, 1.92 [95% CI, 1.03-3.58]; OR for stages I vs II, 0.47 [95% CI, 0.40-0.54]), have high-grade disease (OR, 1.30 [95% CI, 1.09-1.54]), live in a zip code with low educational attainment (OR, 1.19 [95% CI, 1.01-1.42]), and have a higher comorbidity score (OR, 1.59 [95% CI, 1.24-2.03]). Symptomatic tumors were less likely to have lobular vs ductal histologic findings (OR, 0.76 [95% CI, 0.60-0.98]) or to have been diagnosed in 2000 or later (OR, 0.44 [95% CI, 0.38-0.51]) (eTable 2 in the Supplement).

### SEER-Medicare Molecular Cohort: Patient Characteristics and Comparison by Screening Status

**Figure 2.**
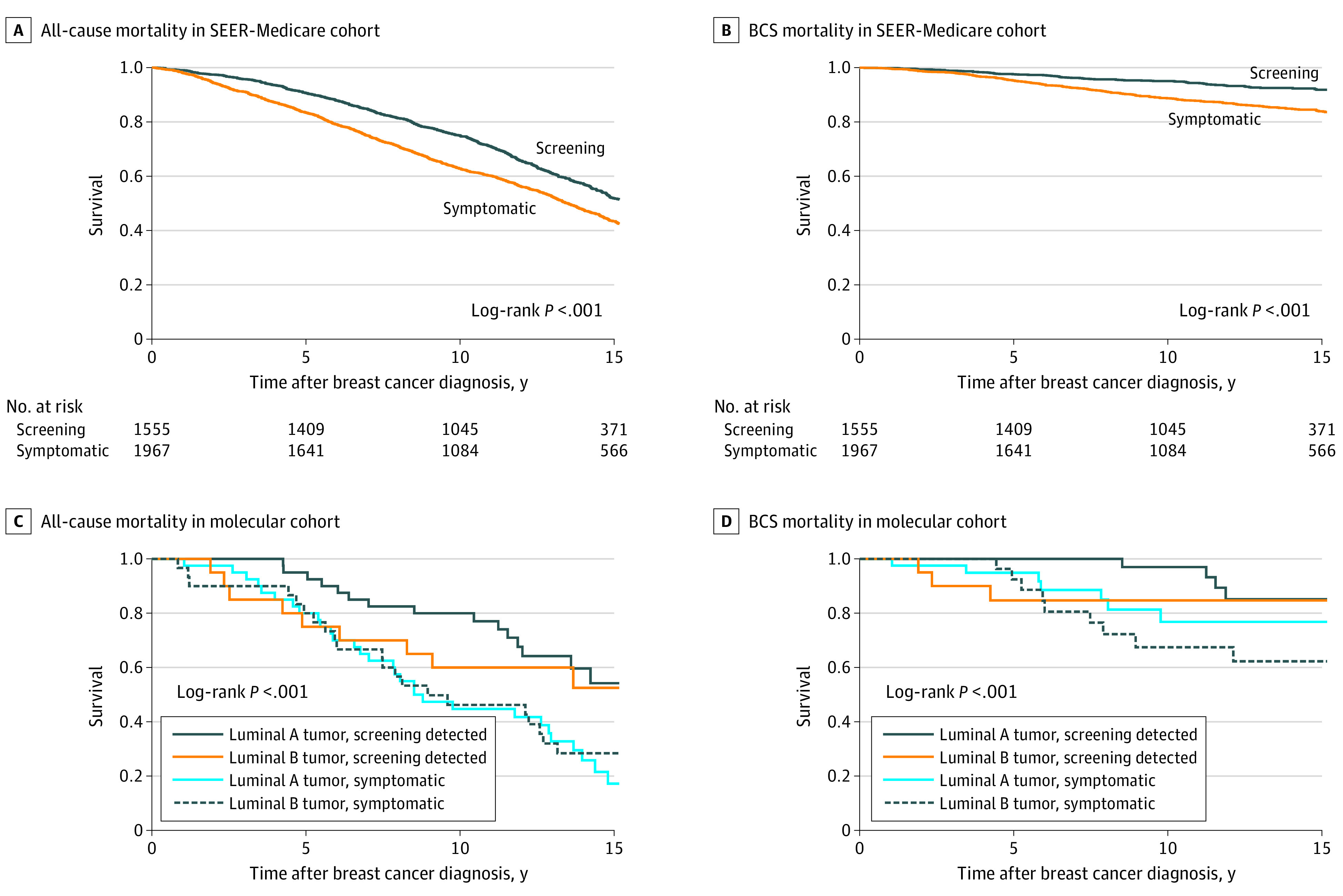
Product Limit Survival Analysis as a Function of Screening vs Symptomatic Tumor Detection Within the Surveillance, Epidemiology, and End Results (SEER)–Medicare cohort (N = 3522), all-cause mortality and breast cancer–specific (BCS) mortality were significantly higher in patients whose tumors were symptomatic. Within the molecular cohort (n = 130), screening detection status and molecular subtype were associated with all-cause mortality but not BCS mortality. The number of patients at risk are censored at 75% of patients for all panels but are not shown for the molecular cohort owing to standard SEER-Medicare data use agreements limiting reporting of cell sizes of fewer than 11.

The molecular cohort consisted of women with tissue blocks pulled for molecular analysis, stratified for relatively equal representation of screen-detected vs symptomatic tumors. RNA quality assurance passed for 97% of samples. Of these, fewer than 11 samples were found to have molecular subtypes of basallike or *ERBB2*-enriched and were excluded from the study, leaving a total of 130 patients for the final analysis with a relatively even distribution of screening-detected (n = 60) vs symptomatic (n = 70) tumors (eTable 3 in the Supplement). As expected, symptomatic tumors had a higher prevalence of T2 tumors (44 of 70 [62.9%] vs 11 of 60 [18.3%]) and older patients (48 of 70 [68.6%] vs 28 of 60 [46.7%] aged 71-75 years) compared with screening-detected tumors. Adjusted analyses confirmed that symptomatic tumors were more likely to be T2 vs T1 (OR, 14.40 [95% CI, 4.86-42.50]) and less likely to have been diagnosed in 2000 or later (OR, 0.16 [95% CI, 0.06-0.45]) (eTable 4 in the Supplement). In unadjusted survival analysis comparing all-cause mortality, patients with screening-detected tumors showed superior overall survival to symptom-detected tumors in both luminal A (77.0% vs 41.7% alive at 10 years) and luminal B (60.0% vs 46.2% alive at 10 years) subtypes (*P* = .008) (Figure 2).

### SEER-Medicare Molecular Cohort: Genomic Signature Combined Analysis

The final model combining screening, demographic, and clinical data with genomic signatures revealed an increased all-cause mortality associated with transforming growth factor β (TGFβ) pathway activation (HR, 5.61 [95% CI, 1.90-16.60]) and p53 dysregulation (HR, 2.15 [95% CI, 1.20-3.86]) in addition to symptomatic detection (HR, 2.49 [95% CI, 1.19-5.20]); multiple comorbidities (HR, 9.94 [95% CI, 3.16-31.30]); negative, borderline, or missing progesterone receptor status (HR, 2.90 [95% CI, 1.05-7.98]); living in a neighborhood with low educational attainment (HR, 5.17 [95% CI, 2.12-12.60]); T2 vs T1 tumor stage (HR, 4.09 [95% CI, 1.79-9.34]); and being 71 to 75 vs 66 to 70 years of age (HR, 2.42 [95% CI, 1.24-4.73]) (Table 2). Factors associated with lower all-cause mortality included increased activity of androgen receptor signaling (HR, 0.23 [95% CI, 0.12-0.45]), macrophage signaling (HR, 0.20 [95% CI, 0.09-0.45]), cytotoxicity (HR, 0.63 [95% CI, 0.44-0.89]), Treg signature (HR, 0.41 [95% CI, 0.21-0.79]), and *ERBB2* signaling (HR, 0.54 [95% CI, 0.30-0.95]). Luminal B vs A subtype did not confer a significant difference in survival after controlling for these pathways (HR, 1.17 [95% CI, 0.53-2.57]). Breast cancer–specific mortality models did not converge because there were not adequate events for the number of variables.

**Table 2.** Surveillance, Epidemiology, and End Results–Medicare Molecular Cohort: Multivariable-Adjusted Cox Proportional Hazards Regression Model of All-Cause Mortality (n = 130)^a^

Parameter	All-cause mortality, HR (95% CI)
Tumor screening factors	
Symptomatic tumor	2.49 (1.19-5.20)
Stage N2 or greater tumor	3.43 (0.72-16.3)
Stage T2 vs T1 tumor	4.09 (1.79-9.34)
High grade vs low/intermediate grade tumor	0.87 (0.39-1.96)
Sociodemographic factors	
Aged 71-75 vs 66-70 y	2.42 (1.24-4.73)
Patient zip code	
Highest quartile: Black race	0.95 (0.48-1.89)
Highest quartile: less than high school education	5.17 (2.12-12.60)
Highest quartile: low-income household	1.21 (0.53-2.74)
Married	1.64 (0.83-3.26)
Lives in metropolitan region	0.84 (0.40-1.73)
Lives in rural area	0.76 (0.18-3.26)
Hawaii tumor registry	0.74 (0.23-2.40)
Clinical factors	
Diagnosed 2000 or later	1.16 (0.55-2.47)
PGR borderline/negative/missing vs positive	2.90 (1.05-7.98)
Subtype luminal B vs luminal A	1.17 (0.53-2.57)
Comorbidity score	
0	1 [Reference]
1	1.29 (0.54-3.07)
≥2	9.94 (3.16-31.30)
Gene signatures	
*ESR1* signature	0.82 (0.51-1.32)
*PGR* signature	0.82 (0.67-1.00)
*ERBB2* signature	0.54 (0.30-0.95)
*FOXA1* signature	0.98 (0.38-2.54)
Androgen receptor signature	0.23 (0.12-0.45)
*IDO1* signature	0.80 (0.42-1.52)
*PDL1* signature	0.90 (0.39-2.03)
*PDL2* signature	1.24 (0.54-2.86)
*PD1* signature	1.63 (0.79-3.36)
*B7_H3* signature	0.95 (0.33-2.76)
* TIGIT*	1.93 (0.96-3.87)
TGFβ signature	5.61 (1.90-16.60)
Endothelial cells signature	1.04 (0.40-2.75)
Macrophage signature	0.20 (0.09-0.45)
Mast cells signature	0.93 (0.69-1.27)
Treg signature	0.41 (0.21-0.79)
BC proliferation signature	1.06 (0.85-1.34)
BC stroma signature	0.75 (0.43-1.32)
APM signature	1.24 (0.73-2.12)
BC cytotoxicity signature	0.63 (0.44-0.89)
BC apoptosis signature	1.09 (0.88-1.34)
BC inflammatory chemokines	1.00 (0.68-1.48)
p53 Dysregulation	2.15 (1.20-3.86)
ER signaling signature	1.11 (0.60-2.07)
Differentiation signature	2.09 (0.65-6.65)
BRCAness signature^b^	0.54 (0.28-1.03)

Abbreviations: APM, antigen processing machinery; BC, breast cancer; ER, estrogen receptor; HR, hazard ratio; PGR, progesterone receptor; TGFβ, transforming growth factor β.

^a^ Colinear signatures (variance inflation factor of >10) were dropped, including claudin low, tumor in situ, CD8 T cells, and cytotoxic T cells.

^b^ Defined by the Breast Cancer 360 NanoString panel.

### Individual Gene-Level Analyses Associated With Screening and Disease Progression

**Figure 3.**
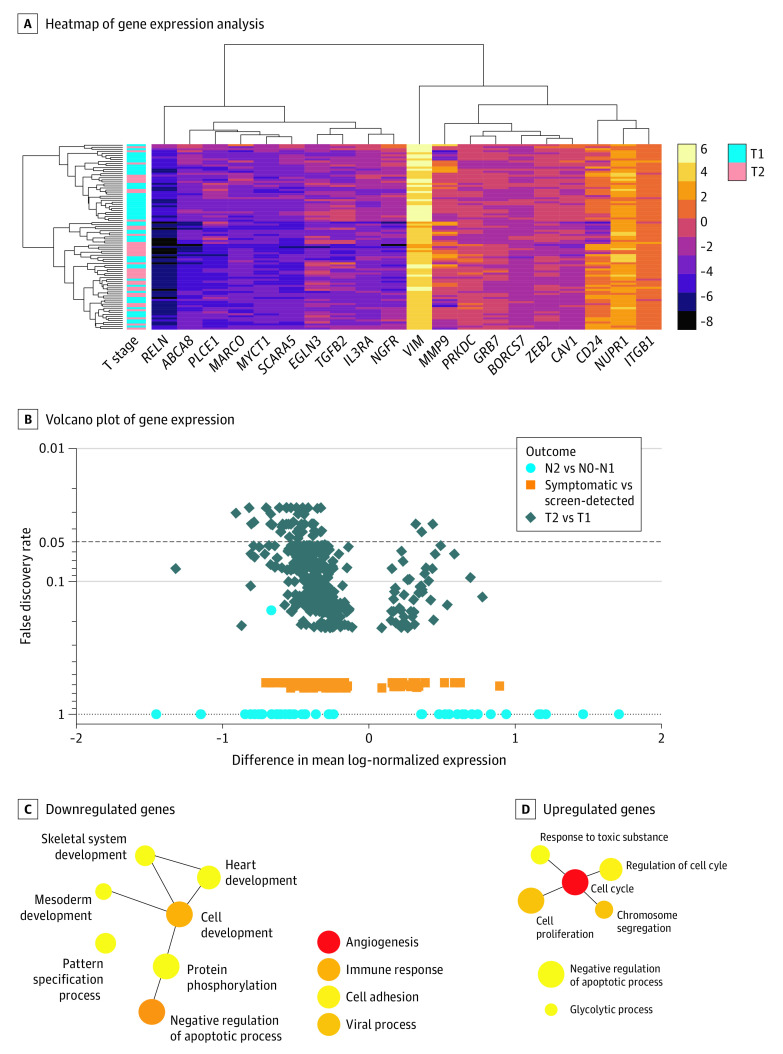
Association of Tumor Size (T2 vs T1) With Differential Gene Expression After Adjusting for Screening Detection and Clinical, Socioeconomic, and Demographic Factors A, Heatmap of NanoString gene expression analysis by top 20 genes associated with symptomatic detection vs screening. B, Volcano plot of differential expression of individual genes (n = 752) in multivariable analysis. Network diagrams of PANTHER, version 16.0, gene ontologies for individual genes expressed in T2 vs T1 tumors are separated by (C) downregulated genes (n = 224) and (D) upregulated genes (n = 29), with circle sizes corresponding to the number of genes within each ontology. Samples with basallike or *ERBB2* subtype cells were excluded from final analysis, leaving an analytic sample of 130 patients.

Increased expression of 95 genes was associated with BCS mortality (Figure 3A and eAppendix in the Supplement). The top differentially expressed genes (unadjusted *P* ≤ .001) were all upregulated and included *KIFC1* (OMIM 603763), *FAM83D* (OMIM 618380), *UBE2C* (OMIM 605574), *CLDN4* (OMIM 602909), *GRB7* (OMIM 601522), and *PKMYT1* (OMIM 602474), each of which have previously reported roles in breast cancer progression. *KIFC1* and *FAM83D* maintained a false discovery rate of 0.05 after multiple hypothesis correction.

The largest differences in gene expression were observed when comparing T2 vs T1 tumors (253 genes), in which 48 genes maintained a false discovery rate of less than 0.05 (Figure 3B). Downregulated genes (n = 224) were enriched for cellular differentiation, immune response, cell adhesion, and regulation of apoptosis (Figure 3C). Upregulated genes (n = 29) were enriched for cell cycle and proliferation, glycolytic metabolism, and regulation of apoptosis (Figure 3D). Only 46 genes were differentially expressed between symptomatic- vs screening-detected tumors and 13 genes between stages III vs II disease (Figure 3B).

### Exhibition of Different Changes in Gene Expression by T Stage in Luminal A and B Tumors

We next hypothesized that T2 vs T1 changes in gene expression would differ by luminal A (88 genes) vs luminal B (100 genes) molecular subtypes owing to distinct mechanisms of disease progression. Only 2 genes were similarly differentially expressed in both subtypes (*FHL1* [OMIM 300163] and *DTX1* [OMIM 602582]). Tumor progression for luminal B, but not luminal A, molecular subtypes was enriched for downregulation of several interferon γ signaling and major histocompatibility complex class II receptor genes. Within a previously published 10-gene signature of interferon regulation,^27^ 8 genes were represented on the BC360 panel, 4 of which were significantly downregulated within T2 vs T1 tumors with luminal B molecular subtypes (*CXCL9* [OMIM 601704], *CCR5* [OMIM 601373], *GZMA* [OMIM 140050], and *HLA-DRA* [OMIM 142860]), with the remaining 4 genes all having numerically decreased expression. Furthermore, 5 of the top 20 differentially expressed genes were *MHCII HLA* genes, with 8 *MHCII HLA* genes being downregulated (*HLA_DMA* [OMIM 142920], *HLA_DMB* [OMIM 142855], *HLA_DPA1* [OMIM 142880], *HLA_DPB1* [OMIM 142858], *HLA_DRA* [OMIM 142860], *HLA_B* [OMIM 142830], *HLA_C* [OMIM 142840], and *HLA_E* [OMIM 143010]) and none being upregulated. In contrast, T2 vs T1 tumors with luminal A molecular subtypes showed no differential expression of interferon γ pathway genes or human leukocyte antigen molecules, and instead showed downregulation of a distinct set of cytokines typically involved in attracting infiltrating immune cells interleukin 6 and 8 and *SDF1* (OMIM 600835).

## Discussion

This study reports the first linkage connecting tumor-based genomic analyses with Medicare administrative claims and SEER clinical, sociodemographic, and vital status data. Using this population-level data set, we were able to model the interaction between screening-based breast cancer detection and sociodemographic characteristics, disease stage, and biological pathway activity as well as their association with overall and BCS mortality. Even after correcting for all clinical and genomic factors, living in a zip code with a poor level of educational attainment remained one of the factors most strongly associated with increased all-cause mortality. Genomic activation of TGFβ and p53 pathways showed adverse associations with survival, whereas improved overall survival was associated with androgen receptor signaling, macrophage infiltration, and activation of cytotoxic T cells. T stage demonstrated the strongest association with changes in gene expression, with other factors such as screening status or N stage showing no associations with gene expression when accounting for T stage. Interestingly, genomic dysregulation associated with T stage differed within luminal A vs B tumors, with luminal B molecular subtype tumors associated with distinct inhibition of interferon γ signaling and *MHCII* expression that was not observed in the luminal A molecular subtype, which instead was associated with cytokine-based immune dysregulation. This study serves as proof-of-principle that combining health service, clinical, sociodemographic, and genomic data together with a single population-level cohort is feasible and may offer new insights into disease progression and factors driving adverse outcomes.

Genomic findings were consistent with our current understanding of the biology of breast cancer, including an adverse association between TGFβ and p53 signaling and a favorable association with androgen receptor signaling and immune infiltration, particularly macrophages and cytotoxic T cells. Differences in immune dysregulation in the progression from T1 to T2 tumors within luminal B vs A molecular subtype tumors may have prognostic or therapeutic implications in tumor immunotherapy. In support of the external validity of our analysis, we observed an adverse outcome associated with increased expression of several genes associated with breast cancer mortality that have been confirmed previously in the literature (*KIFC1*,^28^ *FAM83D*,^29^ *GRB7*,^30^ *UBE2C*,^31^ and *CLDN4*^32^).

An encouraging next-generation iteration of the SEER-RTR concept is the SEER virtual tissue repository (SEER-VTR), which has been implemented recently in 7 SEER registries, including Iowa, Hawaii, Kentucky, Louisiana, Los Angeles, Greater California, and Connecticut. The SEER-VTR works by using SEER-based records to link to the location of tumor blocks stored within community pathology laboratories, which are required by the College of American Pathologists to keep tumor blocks for 10 years after a cancer diagnosis. Prospective partnerships between SEER registries and their community partners can thereby be leveraged to include the physical use of patient samples for anyone diagnosed within the past 10 years. Analogous approach to the one we report in this study using SEER-RTR could be used in collaboration with the SEER-VTR program in future research.

### Limitations 

There are several limitations of this study, including the retrospective and historical nature of our cohort, which did not likely undergo modern imaging, treatment, or genomic risk score profiling. The molecular cohort was limited by small sample sizes owing to the pilot nature of the study. We were unable to assess prescription of nonintravenous medications, including hormonal therapy, which was not available within the Medicare claims data until the introduction of part D in 2006. Many factors known to influence breast cancer could not be incorporated into the study design, including family history and lifestyle factors such as diet, obesity, smoking, and alcohol consumption. Many forms of biological dysregulation were not represented in this study, including somatic^33^ and tumoral mutations, epigenetic changes,^34^ genomic instability, hormonal signaling,^35^ metabolism,^36^ tumor microenvironment,^37^ proteomics, and more, owing to cost and logistic constraints. Instead, we included only a single genomic platform and women with ER-positive tumors to focus on demonstrating the feasibility of linking of genomic, health services, and clinical data together in a single data source and model. Tissue analysis was limited to FFPE, given the archival nature of the specimens. The Iowa and Hawaii populations were unable to be used to analyze representative numbers of Black women. However, the creation of a more racially diverse study cohort is a priority and a topic of active future investigation due to known associations between race and breast tumor biology and molecular subtype.^38,39,40,41^

## 

## Conclusions

By linking SEER-Medicare data to physical tumor specimens, additional connections may be revealed among biology, access to health care, and disparities in breast cancer outcomes. The findings of this population-based cohort study suggest that tumor screening and socioeconomic status are associated with survival in patients who have locally advanced, ER-positive tumors, even when clinical and genomic factors are incorporated. Preliminary analyses suggest that luminal A and B molecular subtypes may be associated with distinct mechanisms of genomic progression when detected at later tumor stages within population-level cohorts.

## Notes

Supplement. **eTable 1.** Characteristics of SEER-Medicare Cohort by Screening Status (N = 3522) **eTable 2.** Patient Characteristics Associated With Having a Symptomatic Tumor vs a Screening-Detected Tumor (N = 3522) **eTable 3.** Baseline Characteristics of All Patients With Tumor Samples Passing QC and Included in Molecular Analysis (n = 130) **eTable 4.** Patient Characteristics Associated With Having a Symptomatic Tumor vs a Screening-Detected Tumor Among Molecular Cohort Patients (n = 130) **eFigure 1.** Diagram SEER-Medicare-RTR Linkage, Case Selection, and Tumor Retrieval **eFigure 2.** Proportion of Screen-Detected Tumors by Year of Diagnosis (N = 3522) **eAppendix.** Gene Expression Data and Survival Analyses
