# Supplementary material for: Analysis of Sociodemographic, Clinical, and Genomic Factors Associated With Breast Cancer Mortality in the Linked Surveillance, Epidemiology, and End Results and Medicare Database
Source: JAMA Netw Open. 2021 Oct 29;4(10):e2131020. doi: 10.1001/jamanetworkopen.2021.31020 (PMC8556625; doi:10.1001/jamanetworkopen.2021.31020)
Supplement: Supplement. — eTable 1. Characteristics of SEER-Medicare Cohort by Screening Status (N = 3522) eTable 2. Patient Characteristics Associated With Having a Symptomatic Tumor vs a Screening-Detected Tumor (N = 3522) eTable 3. Baseline Characteristics of All Patients With Tumor Samples Passing QC and Included in Molecular Analysis (n = 130) eTable 4. Patient Characteristics Associated With Having a Symptomatic Tumor vs a Screening-Detected Tumor Among Molecular Cohort Patients (n = 130) eFigure 1. Diagram SEER-Medicare-RTR Linkage, Case Selection, and Tumor Retrieval eFigure 2. Proportion of Screen-Detected Tumors by Year of Diagnosis (N = 3522) eAppendix. Gene Expression Data and Survival Analyses [file jamanetwopen-e2131020-s001.pdf]

Supplementary Online Content

Robinson TJ, Wilson LE, Marcom PK, et al. Analysis of sociodemographic, clinical, and genomic factors associated with breast cancer mortality in the linked Surveillance, Epidemiology, and End Results and Medicare database. *JAMA Netw Open*. 2021;4(10):e2131020. doi:10.1001/jamanetworkopen.2021.31020

eTable 1. Characteristics of SEER-Medicare Cohort by Screening Status (N = 3522)
eTable 2. Patient Characteristics Associated With Having a Symptomatic Tumor vs a Screening-Detected Tumor (N = 3522)
eTable 3. Baseline Characteristics of All Patients With Tumor Samples Passing QC and Included in Molecular Analysis (n = 130)
eTable 4. Patient Characteristics Associated With Having a Symptomatic Tumor vs a Screening-Detected Tumor Among Molecular Cohort Patients (n = 130)
eFigure 1. Diagram SEER-Medicare-RTR Linkage, Case Selection, and Tumor Retrieval
eFigure 2. Proportion of Screen-Detected Tumors by Year of Diagnosis (N = 3522)
eAppendix. Gene Expression Data and Survival Analyses

This supplementary material has been provided by the authors to give readers additional information about their work.

© 2021 Robinson TJ et al. *JAMA Network Open*.

eTable 1. Characteristics of SEER-Medicare Cohort by Screening Status (N = 3522)

**Variable**	**Screen-detected**	**Symptomatic**	**p-value**
N	1,555	1,967	
Characteristics			
Age			0.287
66-70	703 (45.2)	854 (43.4)	
71-75	852 (54.8)	1,113 (56.6)	
Detailed Race			**0.022**
Black	<11	<11	
East Asian	176 (11.3)	162 (8.2)	
Native Hawaiian	26 (1.7)	46 (2.3)	
Other/Unknown	>15	>25	
White	1,328 (85.4)	1,721 (87.5)	
Stage at diagnosis			**<0.001**
I	1,120 (72.0)	1,059 (53.8)	
II	421 (27.1)	863 (43.9)	
III	14 (0.9)	45 (2.3)	
N Stage			**<0.001**
N0	1,265 (81.3)	1,427 (72.5)	
N1	276 (17.7)	495 (25.2)	
N2+	14 (1.0)	45 (2.3)	
SEER Registry			0.125
Hawaii	281 (18.1)	317 (16.1)	
Iowa	1,274 (81.9)	1,650 (83.9)	
T Stage			**<0.001**
T1	1,316 (84.6)	1,314 (66.8)	
T2	239 (15.4)	653 (33.2)	
Histology			0.286
Ductal	1,253 (80.6)	1,621 (82.4)	
Ductal/Other	23 (1.5)	18 (0.9)	
Lobular	173 (11.1)	197 (10.0)	
Lobular/Ductal	106 (6.8)	131 (6.7)	
Grade			**<0.001**
Low (1-2)	1,154 (74.2)	1,230 (62.5)	
High (3)	299 (19.2)	534 (27.1)	
Missing	102 (6.6)	203 (10.3)	
PR Status			0.415
Borderline/Negative	252 (16.2)	350 (17.8)	
Missing	13 (0.8)	19 (1.0)	
Positive	1,290 (83.0)	1,598 (81.2)	
Tumor size			**<0.001**
5-10 mm	653 (42.0)	486 (24.7)	
10-20 mm	658 (42.3)	822 (41.8)	
20-30 mm	181 (11.6)	457 (23.2)	
30+ mm	63 (4.1)	202 (10.3)	

© 2021 Robinson TJ et al. *JAMA Network Open*.

Comorbidity score			**0.005**
0	1,045 (67.2)	1,258 (64.0)	
1	383 (24.6)	485 (24.6)	
2+	127 (8.2)	224 (11.4)	
Hyperlipidemia	556 (35.8)	504 (25.6)	**<0.001**
Hypertension	777 (50.0)	855 (43.5)	**<0.001**
Diabetes	248 (15.9)	332 (16.9)	0.460
Zip code: 4th QRTL % no HS	321 (20.6)	567 (28.8)	**<0.001**
Zip code: 4th QRTL % black race	405 (26.0)	465 (23.6)	0.100
Zip code: 4th QRTL % living below poverty line	393 (25.3)	477 (24.2)	0.415
Married	973 (62.6)	1,143 (58.1)	**0.007**
Lives in metropolitan area	801 (51.5)	910 (46.3)	**0.002**
Lives in rural area	95 (6.1)	150 (7.6)	0.070
Year of breast cancer dx			**<0.001**
Before 2000	592 (38.1)	1,167 (59.3)	
2000 or later	963 (61.9)	800 (40.7)	

© 2021 Robinson TJ et al. *JAMA Network Open*.

eTable 2. Patient Characteristics Associated With Having a Symptomatic Tumor vs a Screening-Detected Tumor (N = 3522)

Patient characteristics	*Unadjusted OR*	*Multivariate-adjusted OR*
Age at dx (ref = 66-70)		
71-75	1.08 (0.94, 1.23)	1.05 (0.91, 1.21)
Stage at diagnosis (ref=II)		
I	**0.46 (0.40, 0.53)**	**0.47 (0.40, 0.54)**
III	1.57 (0.85, 2.89)	**1.92 (1.03, 3.58)**
Grade (ref=Low)		
High	**1.68 (1.42, 1.97)**	**1.30 (1.09, 1.54)**
Missing	**1.87 (1.45, 2.40)**	**1.53 (1.15, 2.02)**
Black race (ref= other than Black)	1.42 (0.48, 4.26)	-
Detailed race (ref= White)		
Black	1.23 (0.40, 3.78)	1.19 (0.37, 3.86)
East Asian	**0.71 (0.57, 0.89)**	0.80 (0.62, 1.02)
Native Hawaiian	1.37 (0.84, 2.22)	1.50 (0.90, 2.51)
Other	1.16 (0.65, 2.05)	1.23 (0.67, 2.24)
Comorbidity score (ref=0)		
1	1.05 (0.90, 1.23)	1.06 (0.90, 1.26)
2+	**1.47 (1.16, 1.85)**	**1.59 (1.24, 2.03)**
Zip code at diagnosis		
Highest quartile- black race	0.88 (0.75, 1.03)	1.02 (0.86, 1.22)
Highest quartile- did not finish high school	**1.56 (1.33, 1.82)**	**1.19 (1.01, 1.42)**
Highest quartile- household income below poverty line	0.95 (0.81, 1.10)	0.91 (0.77, 1.09)
Married	**0.83 (0.72, 0.95)**	0.89 (0.77, 1.03)
Lives in metropolitan area	**0.81 (0.71, 0.93)**	0.90 (0.76, 1.05)
Lives in rural area	1.27 (0.97, 1.66)	1.19 (0.89, 1.59)
Histology (ref=Ductal)		
Ductal/other	0.60 (0.33, 1.13)	0.87 (0.46, 1.66)
Lobular	0.88 (0.71, 1.09)	**0.76 (0.60, 0.98)**
Lobular/Ductal	0.96 (0.73, 1.25)	1.02 (0.77, 1.35)
PR Status (ref=Positive)		
Borderline/Negative	1.12 (0.94, 1.34)	1.07 (0.89, 1.29)
Missing	1.18 (0.58, 2.40)	0.92 (0.44, 1.91)
Year of diagnosis (reference=before 2000)		
2000 or later	**0.42 (0.37, 0.48)**	**0.44 (0.38, 0.51)**

© 2021 Robinson TJ et al. *JAMA Network Open*.

eTable 3. Baseline Characteristics of All Patients With Tumor Samples Passing QC and Included in Molecular Analysis (n = 130)

**Variable**	**Screen-detected**	**Symptomatic**	**p-value**
N	60	70	
Characteristics			
Age			**0.011**
66-70	32 (53.3)	22 (31.4)	
71-75	28 (46.7)	48 (68.6)	
Race			0.410
White	49 (81.7)	53 (75.7)	
Black	0	0	
East Asian/Native Hawaiian/Other	11 (18.3)	17 (24.3)	
N Stage N1	50 (83.3)	56 (80.0)	0.801
High histologic grade	16 (26.7)	18 (25.7)	0.299
SEER Registry			0.418
HI	15 (25.0)	22 (31.4)	
IA	45 (75.0)	48 (68.6)	
T Stage			**<0.001**
I	49 (81.7)	26 (37.1)	
II	11 (18.3)	44 (62.9)	
Histology			0.699
Ductal and Ductal/Other	46 (76.7)	54 (77.1)	
Lobular and Lobular/Ductal	14 (23.3)	16 (22.9)	
ER Status			
Positive	60 (100)	70 (100)	
PR Status			0.198
Borderline/Missing/Negative	<11	14 (20)	
Positive	>48	56 (80)	
Tumor size			**<0.001**
5-10 mm	15 (25.0)	<11	
10-20 mm	34 (56.7)	>17	
20+ mm	11 (18.3)	44 (62.8)	
Patient zip code demographics:			
% not finishing high school, Median (Q1, Q3)	14.8 (12.6, 19.3)	13.9 (11.2, 18.8)	0.437
% black race, Median (Q1, Q3)	1.0 (0.2, 2.5)	1.0 (0.3, 2.6)	0.503
% living below poverty line, Median (Q1, Q3)	10.1 (6.7, 12.6)	9.2 (6.0, 12.3)	0.719
Married	36 (60.0)	38 (54.3)	0.512
Lives in metropolitan area	33 (55.0)	44 (62.8)	0.363
Lives in rural area			0.423
No	57 (95.0)	64 (91.4)	
Year of breast cancer dx			<0.001
Pre-2000	19 (31.7)	45 (64.2)	

© 2021 Robinson TJ et al. *JAMA Network Open*.

2000 or later	41 (68.3)	25 (35.7)	
Died of breast cancer within 10 years of diagnosis	17 (28.3)	35 (50.0)	<0.001
Died of any cause within 10 years of diagnosis	24 (40.0)	51 (72.9)	<0.001

© 2021 Robinson TJ et al. *JAMA Network Open*.

eTable 4. Patient Characteristics Associated With Having a Symptomatic Tumor vs a Screening-Detected Tumor Among Molecular Cohort Patients (n = 130)

	Univariable OR	*Multivariable OR*
Stage N2+ vs N1	1.76 (0.31, 9.95)	1.12 (0.10, 12.3)
Stage T2 vs T1	**7.54 (3.34, 17.0)**	**14.4 (4.86, 42.5)**
High grade	0.95 (0.43, 2.08)	0.39 (0.12, 1.27)
Aged 71-76 vs 66-70	2.49 (1.22, 5.10)	**3.27 (1.18, 9.10)**
Comorbidity Score (ref=0)		
1	1.20 (0.48, 2.98)	0.56 (0.15, 2.06)
2+	0.75 (0.25, 2.24)	0.36 (0.07, 1.98)
Patient zip code		
Highest quartile- black race	1.22 (0.58, 2.55)	0.65 (0.22, 1.93)
Highest quartile- < HS education	0.88 (0.40, 1.95)	0.48 (0.13, 1.77)
Highest quartile- households in poverty	0.82 (0.36, 1.85)	1.23 (0.36, 4.28)
Married	0.79 (0.39, 1.59)	0.98 (0.37, 2.59)
Lives in metro region	1.38 (0.69, 2.80)	1.66 (0.53, 5.20)
Lives in rural area	1.78 (0.43, 7.45)	1.57 (0.18, 13.90)
Hawaii Tumor Registry	1.37 (0.64, 2.98)	1.40 (0.43, 4.59)
Other race vs White*	1.43 (0.61, 3.35)	-
PR Borderline/Negative vs Positive	1.89 (0.71, 5.05)	2.23 (0.59, 8.47)
Subtype Luminal B vs Luminal A	1.50 (0.73, 3.07)	1.52 (0.54, 4.25)
Diagnosed in 2000 or later	**0.26 (0.12, 0.53)**	**0.16 (0.06, 0.45)**

*collinear with Hawaii Tumor Registry in this set; excluded from multivariable analysis

© 2021 Robinson TJ et al. *JAMA Network Open*.

eFigure 1. Diagram SEER-Medicare-RTR Linkage, Case Selection, and Tumor Retrieval

SEER-Medicare and Molecular cohort creation by application of inclusion and exclusion criteria. In order to apply inclusion/exclusion criteria that require both 1) SEER-Medicare based data (stage, part A&B coverage, etc.) and 2) tissue availability we used the following approach: **A)** SEER-RTR PIs (Hawaii and Iowa) provided the NCI a list of all SEER IDs for breast cancer patients age 65 or older for which they had tissue. **B)** NCI linked all Iowa and Hawaii SEER patients with Medicare data for any patients with Medicare enrollment and created an encrypted SEER-Medicare ID for each beneficiary, with a crosswalk file provided to each registry in order to be able to link encrypted SEER-Medicare IDs with specific SEER cases. **C)** After the encrypted SEER-Medicare data were received by the research team, our team applied inclusion/exclusion criteria and selected a subset of patients that have available tissues, stratified to ensure a roughly equal mix of patients with screening vs. non-screening detected tumors. This list included more than the number of samples we ultimately anticipated processing, as it was presumed that some banked samples might not have adequate tissue amount or quality for analysis when pulled and examined by the SEER-RTR PIs. **D)** A list of requested samples indexed by encrypted SEER-Medicare ID was sent to the NCI. **E)** The NCI provided a crosswalk file to link SEER IDs with encrypted SEER-Medicare IDs. **F)** The SEER-RTR PIs pulled corresponding samples and sent them to the research team labeled with the encrypted SEER-Medicare IDs. This approach allowed pulling samples stratified by the key independent variable of interest (screening vs. clinically detected (i.e. non-screened) cancers and avoided unnecessary and costly pulling of tumor blocks that would not be used in the final analysis.

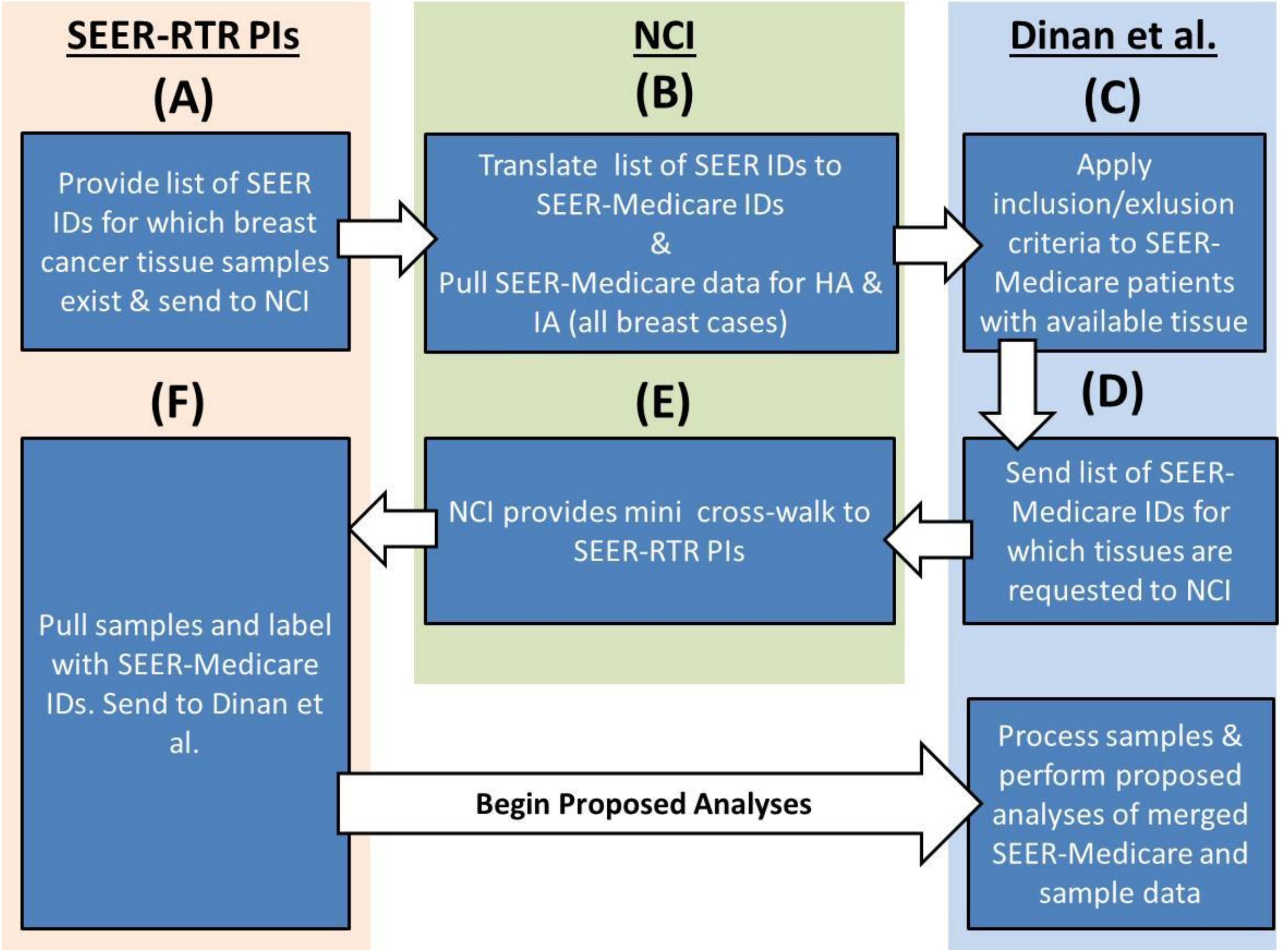

© 2021 Robinson TJ et al. *JAMA Network Open*.

eFigure 2. Proportion of Screen-Detected Tumors by Year of Diagnosis (N = 3522)

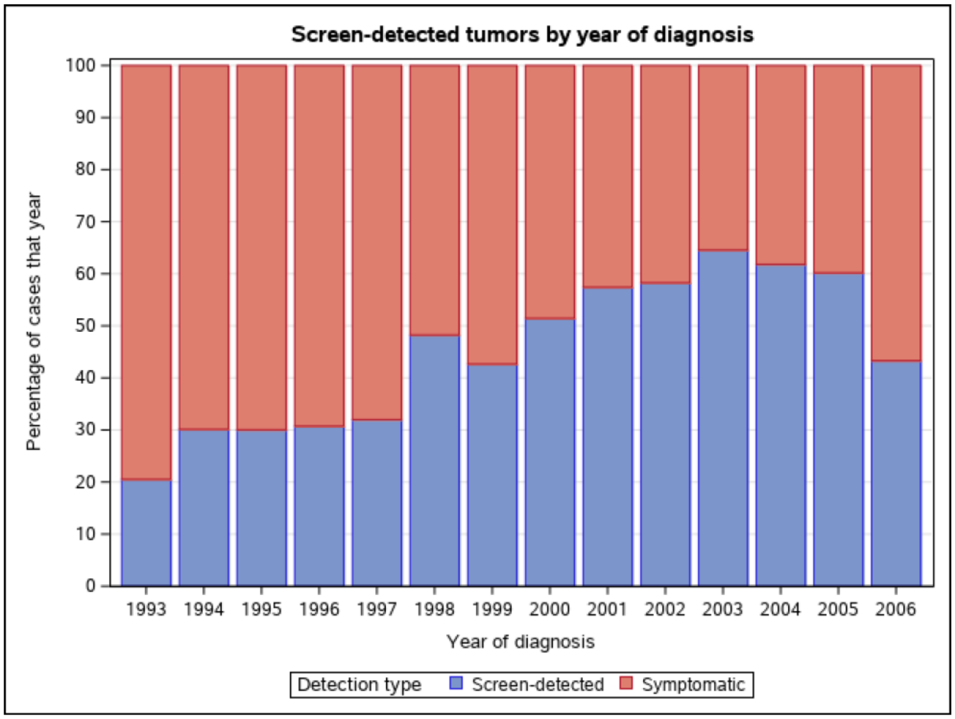

© 2021 Robinson TJ et al. *JAMA Network Open*.

eAppendix. Gene Expression Data and Survival Analyses

Overall Survival – Genes with p ≤ 0.05 shown for all Tables below.

Survival Analyses (Overall Survival & Breast Cancer-Specific Survival): Multivariable expression analysis of genes associated with increased all-cause or breast cancer-specific mortality. Hazard ratios > 1 indicate increased mortality.

Changes in gene expression (linear regression):

Symptomatic vs. Screen-detected disease

N2 vs. N1-0 disease

T2 vs. T1 tumors

T2 vs. T1 limited to Luminal A tumors

T2 vs. T1 limited to Luminal B tumors

List of all genes on the Nanostring platform: An alphabetical list of all 752 gene symbols analyzed by the Nanostring BC360 panel is provided to allow users to know the full set of genes that were investigated.

Multivariable-adjusted Cox PH regression for all-cause mortality and log-normalized gene expression

Obs	*Estimate*	*ProbChiSq*	*HazardRatio*	*gene*	*Hochberg*	*FalseDiscoveryRate*
1	0.3009	0.001	1.351	AREG	0.7883	0.3975
2	-0.94338	0.0012	0.389	PARP4	0.8891	0.3975
3	1.26123	0.0016	3.53	NCAPH2	0.998	0.3975
4	0.80401	0.0025	2.234	MCM2	0.998	0.4757
5	-0.79438	0.0032	0.452	RBL2	0.998	0.486
6	1.1461	0.0039	3.146	PSMB7	0.998	0.49
7	0.61405	0.0056	1.848	UBE2C	0.998	0.5415
8	-0.44321	0.0058	0.642	WNT2	0.998	0.5415
9	0.45573	0.0092	1.577	ALDOA	0.998	0.5984

© 2021 Robinson TJ et al. *JAMA Network Open*.

10	-0.52366	0.01	0.592	AR	0.998	0.5984
11	-0.4145	0.0104	0.661	HLA_DOB	0.998	0.5984
12	0.34934	0.0125	1.418	PKMYT1	0.998	0.5984
13	-0.39303	0.0139	0.675	ALDH1A1	0.998	0.5984
14	-0.68016	0.0148	0.507	TLR4	0.998	0.5984
15	1.03315	0.0157	2.81	MCM3	0.998	0.5984
16	0.53722	0.0158	1.711	CDC7	0.998	0.5984
17	0.47037	0.0158	1.601	RRM2	0.998	0.5984
18	0.54111	0.0164	1.718	UBE2T	0.998	0.5984
19	0.48087	0.0165	1.617	TYMS	0.998	0.5984
20	-0.67349	0.0167	0.51	ATM	0.998	0.5984
21	0.50151	0.0185	1.651	CXXC5	0.998	0.5984
22	-0.48306	0.0189	0.617	TUBA4A	0.998	0.5984
23	-0.56197	0.0202	0.57	IL6R	0.998	0.5984
24	-0.80032	0.0206	0.449	TAPBP	0.998	0.5984
25	0.29513	0.0215	1.343	ST6GALNAC2	0.998	0.5984
26	-0.46994	0.022	0.625	PIK3R5	0.998	0.5984
27	-0.239	0.0222	0.787	ZBTB16	0.998	0.5984
28	0.42061	0.0234	1.523	KIFC1	0.998	0.5984
29	-0.45293	0.0236	0.636	PIK3CG	0.998	0.5984
30	-0.31578	0.024	0.729	HLA_DRA	0.998	0.5984
31	-0.61392	0.0247	0.541	FLI1	0.998	0.5984
32	0.50417	0.0267	1.656	CCNB1	0.998	0.61
33	0.4097	0.0268	1.506	CDC25A	0.998	0.61
34	-0.28751	0.0301	0.75	IL7R	0.998	0.666
35	0.34719	0.0319	1.415	EXO1	0.998	0.6811
36	-0.24081	0.033	0.786	ASPN	0.998	0.6811
37	0.40164	0.0343	1.494	MT1G	0.998	0.6811

© 2021 Robinson TJ et al. *JAMA Network Open*.

38	0.28045	0.0344	1.324	CCND1	0.998	0.6811
39	-0.46792	0.0383	0.626	CBLC	0.998	0.7058
40	0.33656	0.0392	1.4	TTK	0.998	0.7058
41	0.20586	0.0392	1.229	DUSP4	0.998	0.7058
42	-0.48679	0.0408	0.615	NPEPPS	0.998	0.7058
43	-0.40428	0.0418	0.667	HLA_DMA	0.998	0.7058
44	0.50497	0.0425	1.657	GRB2	0.998	0.7058
45	-0.27047	0.0431	0.763	FLT3	0.998	0.7058
46	0.3777	0.0439	1.459	HELLS	0.998	0.7058
47	-0.24177	0.0462	0.785	CCL3L1	0.998	0.7058
48	0.17817	0.0463	1.195	FGFR4	0.998	0.7058
49	-0.39093	0.0463	0.676	BNC2	0.998	0.7058
50	-0.25611	0.0469	0.774	CCL4	0.998	0.7058
51	-0.28218	0.048	0.754	FGL2	0.998	0.7083
52	-0.3843	0.0498	0.681	HIF1A	0.998	0.7203

© 2021 Robinson TJ et al. *JAMA Network Open*.

Breast Cancer (BC) Specific Survival – Genes with p ≤ 0.05 shown

Obs	*Estimate*	*ProbChiSq*	*HazardRatio*	*gene*	*Hochberg*	*FalseDiscoveryRate*
1	1.77738	<.0001	5.914	KIFC1	0.0551	0.0522
2	1.87014	0.0001	6.489	FAM83D	0.1042	0.0522
3	1.80857	0.0003	6.102	UBE2C	0.2343	0.0625
4	1.31367	0.0004	3.72	CLDN4	0.3066	0.0625
5	1.75	0.0004	5.755	GRB7	0.311	0.0625
6	0.95237	0.0009	2.592	PKMYT1	0.6714	0.1005
7	1.42166	0.001	4.144	POLQ	0.7772	0.1005
8	1.57382	0.0011	4.825	RRM2	0.7964	0.1005
9	0.63664	0.0015	1.89	ZIC2	0.9995	0.1253
10	0.82158	0.0017	2.274	MYBL2	0.9995	0.1253
11	0.99747	0.0018	2.711	FOXM1	0.9995	0.1253
12	1.06974	0.0022	2.915	NSD3	0.9995	0.1282
13	1.64817	0.0022	5.197	MCM2	0.9995	0.1282
14	-1.3627	0.0024	0.256	MSR1	0.9995	0.1282
15	1.85766	0.0029	6.409	CCNB1	0.9995	0.1413
16	1.02994	0.0034	2.801	CEP55	0.9995	0.1413
17	1.13513	0.0036	3.112	RAD51	0.9995	0.1413
18	0.89656	0.0039	2.451	CDC25C	0.9995	0.1413
19	-1.60656	0.0039	0.201	PARP4	0.9995	0.1413
20	1.29396	0.0039	3.647	TOP2A	0.9995	0.1413
21	1.36824	0.0039	3.928	BAIAP2L1	0.9995	0.1413
22	0.92504	0.0046	2.522	TTK	0.9995	0.1523
23	0.7774	0.0051	2.176	EXO1	0.9995	0.1523
24	1.56106	0.0053	4.764	HIST1H1C	0.9995	0.1523
25	0.89081	0.0057	2.437	E2F1	0.9995	0.1523
26	0.94697	0.0058	2.578	ESPL1	0.9995	0.1523

© 2021 Robinson TJ et al. *JAMA Network Open*.

27	0.9089	0.0063	2.482	ORC6	0.9995	0.1523
28	1.02729	0.0063	2.793	AURKA	0.9995	0.1523
29	1.13542	0.0065	3.112	MKI67	0.9995	0.1523
30	-0.81176	0.0065	0.444	SFRP2	0.9995	0.1523
31	0.69317	0.0066	2	MELK	0.9995	0.1523
32	-0.95438	0.0067	0.385	FAP	0.9995	0.1523
33	1.0838	0.0067	2.956	CXXC5	0.9995	0.1523
34	0.94427	0.007	2.571	CDCA5	0.9995	0.154
35	1.02732	0.0075	2.794	HMGA1	0.9995	0.1596
36	1.06163	0.0076	2.891	HIST1H3H	0.9995	0.1596
37	1.05946	0.0081	2.885	CDC6	0.9995	0.1652
38	1.84422	0.009	6.323	NUDT1	0.9995	0.1783
39	0.97087	0.0093	2.64	CDKN3	0.9995	0.18
40	1.11839	0.0101	3.06	HIST1H2BH	0.9995	0.1838
41	0.90383	0.0102	2.469	KIF11	0.9995	0.1838
42	1.25001	0.0103	3.49	RFC4	0.9995	0.1838
43	0.57817	0.0107	1.783	NUF2	0.9995	0.1863
44	0.43716	0.0114	1.548	FGFR4	0.9995	0.1864
45	-0.63165	0.0115	0.532	ASPN	0.9995	0.1864
46	0.95586	0.0116	2.601	MAD2L1	0.9995	0.1864
47	1.00259	0.0116	2.725	AURKB	0.9995	0.1864
48	0.60062	0.0134	1.823	BIRC5	0.9995	0.2105
49	0.62537	0.0173	1.869	VEGFA	0.9995	0.2563
50	1.08819	0.0174	2.969	ATAD2	0.9995	0.2563
51	0.89788	0.0178	2.454	RAD54L	0.9995	0.2563
52	1.00366	0.018	2.728	BRCA1	0.9995	0.2563
53	1.94157	0.0184	6.97	RBL1	0.9995	0.2563
54	1.51682	0.0188	4.558	HDAC2	0.9995	0.2563

© 2021 Robinson TJ et al. *JAMA Network Open*.

55	1.23554	0.019	3.44	MIS18A	0.9995	0.2563
56	1.35955	0.0194	3.894	DDX39A	0.9995	0.2563
57	0.80633	0.0197	2.24	CDC25A	0.9995	0.2563
58	0.90425	0.0198	2.47	SPC25	0.9995	0.2563
59	0.60807	0.0229	1.837	ELF3	0.9995	0.292
60	0.733	0.0242	2.081	KIF14	0.9995	0.3011
61	1.13335	0.0246	3.106	SUV39H2	0.9995	0.3011
62	-1.43438	0.0248	0.238	ADD1	0.9995	0.3011
63	0.88361	0.0255	2.42	CENPF	0.9995	0.304
64	0.78402	0.026	2.19	KIF2C	0.9995	0.3052
65	-0.58613	0.0283	0.556	HLA_DPB1	0.9995	0.3271
66	0.81839	0.0298	2.267	ANLN	0.9995	0.3391
67	0.66298	0.0319	1.941	EIF4EBP1	0.9995	0.3509
68	0.80687	0.0319	2.241	NDC80	0.9995	0.3509
69	0.97587	0.0322	2.653	SLC2A1	0.9995	0.3509
70	-0.89322	0.0335	0.409	AR	0.9995	0.3601
71	-0.58506	0.0349	0.557	HLA_DRA	0.9995	0.3684
72	0.84182	0.0353	2.321	ID2	0.9995	0.3684
73	-0.63214	0.0359	0.531	ALDH1A1	0.9995	0.3697
74	-1.43528	0.0368	0.238	JAK1	0.9995	0.3725
75	-0.9599	0.0375	0.383	LRRC32	0.9995	0.3725
76	0.76908	0.0386	2.158	ASPM	0.9995	0.3725
77	0.66593	0.0387	1.946	POLD1	0.9995	0.3725
78	0.594	0.039	1.811	NEIL3	0.9995	0.3725
79	-0.79756	0.0391	0.45	TUBA4A	0.9995	0.3725
80	0.64688	0.0402	1.91	HIST3H2BB	0.9995	0.3776
81	0.86112	0.0419	2.366	CCNE2	0.9995	0.3803
82	-0.75038	0.0421	0.472	ITGA6	0.9995	0.3803

© 2021 Robinson TJ et al. *JAMA Network Open*.

83	0.55359	0.0424	1.739	EDN1	0.9995	0.3803
84	-0.62066	0.0432	0.538	FAM198B	0.9995	0.3803
85	0.88908	0.0433	2.433	UBE2T	0.9995	0.3803
86	-0.5973	0.0444	0.55	MMP14	0.9995	0.3803
87	-0.41315	0.0447	0.662	ZBTB16	0.9995	0.3803
88	-0.45944	0.0447	0.632	FST	0.9995	0.3803
89	0.91463	0.0451	2.496	CCNA2	0.9995	0.3803
90	-0.79166	0.0457	0.453	COL6A3	0.9995	0.3803
91	0.87758	0.046	2.405	CDC20	0.9995	0.3803
92	-0.65998	0.0478	0.517	TBC1D9	0.9995	0.3904
93	-0.79405	0.0486	0.452	JCAD	0.9995	0.3919
94	0.69837	0.0493	2.01	HELLS	0.9995	0.3919
95	0.67327	0.0498	1.961	BNIP3	0.9995	0.3919
96	-0.7707	0.05	0.463	PIK3R1	0.9995	0.3919

© 2021 Robinson TJ et al. *JAMA Network Open*.

Symptomatic vs. Screen-detected – Genes with p ≤ 0.05 shown

Obs	*Estimate*	*ProbChiSq*	*Gene*	*FDR*
1	0.38524	0.0032	NUPR1	0.5795
2	-0.53802	0.0033	EGLN3	0.5795
3	-0.3511	0.0059	IL3RA	0.5795
4	0.30569	0.0083	PRKDC	0.5795
5	-0.28429	0.0086	ZEB2	0.5795
6	-0.2024	0.0123	BORCS7	0.5795
7	-0.30525	0.0132	MYCT1	0.5795
8	-0.49628	0.0138	RELN	0.5795
9	0.32274	0.0138	GRB7	0.5795
10	-0.56868	0.0151	NGFR	0.5795
11	-0.19413	0.0151	ITGB1	0.5795
12	-0.57363	0.0158	ABCA8	0.5795
13	-0.53322	0.016	PLCE1	0.5795
14	-0.35187	0.0165	CAV1	0.5795
15	-0.70516	0.0171	MMP9	0.5795
16	-0.39561	0.0185	MARCO	0.5795
17	-0.29183	0.0194	VIM	0.5795
18	-0.47678	0.0202	TGFB2	0.5795
19	-0.40802	0.0207	SCARA5	0.5795
20	0.6254	0.0212	CD24	0.5795
21	-0.23047	0.0213	MAF	0.5795
22	-0.29359	0.0216	PDGFRA	0.5795
23	0.23085	0.0217	FOXA1	0.5795
24	-0.32478	0.0218	TGFBR2	0.5795
25	0.30045	0.0226	BAG1	0.5795
26	-0.67423	0.0244	KRT7	0.5795
27	-0.44184	0.0251	FHL1	0.5795
28	-0.58409	0.0283	LEP	0.5795
29	-0.37266	0.0291	LEPR	0.5795
30	-0.23155	0.0303	ACVRL1	0.5795
31	0.15828	0.0305	ACVR1B	0.5795
32	0.23181	0.0306	BBC3	0.5795
33	-0.39449	0.031	DCN	0.5795
34	-0.27359	0.0323	CD34	0.5795
35	-0.31823	0.0328	FSTL1	0.5795
36	-0.28082	0.0328	CXorf36	0.5795
37	-0.3104	0.0343	RAC2	0.5795
38	-0.2626	0.0347	MAML2	0.5795
39	-0.35867	0.0348	GZMH	0.5795
40	-0.64975	0.0358	AGR2	0.5795

© 2021 Robinson TJ et al. *JAMA Network Open*.

41	-0.24961	0.0377	CCND2	0.5795
42	-0.34019	0.0379	PRKCB	0.5795
43	-0.24066	0.0382	SMO	0.5795
44	-0.38023	0.0384	GZMA	0.5795
45	-0.32137	0.0385	FGF7	0.5795
46	-0.27276	0.0397	JCAD	0.5795
47	0.16706	0.0403	SUV39H2	0.5795
48	-0.16607	0.0408	ITGAV	0.5795
49	-0.2618	0.0409	PIK3CG	0.5795
50	-0.22504	0.0423	EPAS1	0.5795
51	0.22331	0.0427	IFT140	0.5795
52	-0.48021	0.0443	ASPN	0.5795
53	-0.47756	0.0443	CD36	0.5795
54	-0.31396	0.0451	DTX1	0.5795
55	-0.31331	0.0464	CCL2	0.5795
56	-0.33955	0.0467	FGL2	0.5795
57	-0.51133	0.0484	SPP1	0.5795
58	-0.58948	0.0485	SCUBE2	0.5795
59	-0.3085	0.049	PPARG	0.5795
60	-0.33366	0.0494	CLEC5A	0.5795
61	-0.32223	0.0497	MMP14	0.5795
62	-0.29661	0.0497	FLNC	0.5795
63	0.51651	0.0498	FUT3	0.5795

© 2021 Robinson TJ et al. *JAMA Network Open*.

N2 vs. N1-0 – Genes with p ≤ 0.05 shown

Obs	*Estimate*	*ProbChiSq*	*Gene*	*FDR*
Obs	*Estimate*	*ProbChiSq*	*HazardRatio*	*gene*
1	-0.66739	0.0002	SMAD5	0.1651
2	-0.73249	0.0079	SERPINH1	0.9998
3	-0.57282	0.0096	PTEN	0.9998
4	1.70934	0.0189	PROM1	0.9998
5	0.64057	0.0202	ERBB2	0.9998
6	-0.24177	0.0219	PRKACA	0.9998
7	-0.43885	0.0236	BMPR1A	0.9998
8	-0.84642	0.0288	PBX3	0.9998
9	-0.66526	0.0291	NSD3	0.9998
10	0.65417	0.0324	HLA_C	0.9998
11	0.60513	0.0401	BLVRA	0.9998
12	1.16188	0.0426	SLPI	0.9998
13	-0.8085	0.0444	OCLN	0.9998
14	1.17493	0.0445	CKMT1A	0.9998
15	0.54845	0.0448	RAC3	0.9998
16	-0.7366	0.0466	GTF2H2	0.9998
17	0.36365	0.0474	BAD	0.9998
18	-0.43461	0.0481	ELK3	0.9998

© 2021 Robinson TJ et al. *JAMA Network Open*.

T2 vs. T1 – Genes with p ≤ 0.05 shown

Obs	*Estimate*	*ProbChiSq*	*Gene*	*FDR*
1	-0.53309	<.0001	NFATC1	0.0279
2	-0.61138	<.0001	PIM1	0.0279
3	-0.56472	0.0003	DTX1	0.0279
4	-0.56196	0.0003	CCL2	0.0279
5	-0.41531	0.0003	RORA	0.0279
6	-0.70251	0.0003	FHL1	0.0279
7	-0.45303	0.0004	PIK3CG	0.0279
8	-0.60237	0.0004	LEPR	0.0279
9	-0.32853	0.0004	ELK3	0.0279
10	-0.49502	0.0004	SPRY2	0.0279
11	-0.81859	0.0005	NGFR	0.0279
12	-0.55592	0.0005	FOSL1	0.0279
13	-0.49395	0.0005	ICAM1	0.0279
14	-0.35242	0.0005	PSMB10	0.0283
15	-0.90901	0.0006	LEP	0.0307
16	-0.48407	0.0007	RUNX3	0.031
17	-0.67117	0.0007	CRYAB	0.031
18	-0.44188	0.0008	CXorf36	0.0314
19	-0.51666	0.0009	PPARG	0.0357
20	-0.78543	0.0009	ABCA8	0.0357
21	-0.80347	0.001	THBS4	0.037
22	-0.58951	0.0011	IGF1	0.037
23	0.32002	0.0012	HDAC2	0.037
24	-0.37565	0.0013	CDC14B	0.037
25	-0.39179	0.0013	PIK3CD	0.037
26	-0.38603	0.0013	CCND2	0.037
27	0.43649	0.0015	CCNB1	0.0371
28	-0.66585	0.0016	F3	0.0371
29	-0.54935	0.0016	EGFR	0.0371
30	-0.6706	0.0016	LPL	0.0371
31	-0.43957	0.0017	ID1	0.0371
32	-0.53283	0.0017	GZMH	0.0371
33	-0.46093	0.0017	CAV1	0.0371
34	-0.56416	0.0017	SOCS3	0.0371
35	-0.38133	0.0017	CDH5	0.0371
36	-0.65904	0.0018	CACNA1D	0.0376
37	-0.59356	0.0019	IL1B	0.0389
38	-0.47797	0.002	BCL2A1	0.0403
39	-0.32229	0.0022	HLA_E	0.0418
40	-0.37134	0.0022	CBLC	0.0418

© 2021 Robinson TJ et al. *JAMA Network Open*.

41	-0.33055	0.0024	ZEB2	0.0424
42	-0.60253	0.0025	CACNA2D3	0.0424
43	-0.49021	0.0025	TWIST2	0.0424
44	-0.45629	0.0025	FLNC	0.0424
45	0.36095	0.0025	ALDOA	0.0424
46	-0.38224	0.0028	LRRC32	0.0452
47	-0.54292	0.0029	COL27A1	0.0463
48	-0.4854	0.003	TGFB3	0.0474
49	-0.49802	0.0035	TSPAN7	0.053
50	-0.30149	0.0035	PLD1	0.053
51	-0.40602	0.0037	S1PR1	0.0538
52	-0.68332	0.0041	CD36	0.0538
53	-0.31768	0.0041	PECAM1	0.0538
54	-0.44288	0.0041	ITGB3	0.0538
55	-0.32579	0.0041	PIK3R1	0.0538
56	0.4907	0.0042	HIST3H2BB	0.0538
57	-0.42438	0.0043	CXCL12	0.0538
58	-0.3537	0.0043	MYCT1	0.0538
59	-0.52145	0.0043	PTGS2	0.0538
60	-0.53059	0.0043	MEOX2	0.0538
61	-0.44651	0.0044	SNAI1	0.0538
62	-0.41411	0.0045	JAM2	0.054
63	-0.38915	0.0046	SPN	0.054
64	-0.78836	0.0047	SFRP1	0.054
65	-0.64358	0.0048	IL6	0.054
66	-0.499	0.0049	SCARA5	0.054
67	-0.28689	0.0049	TGFB1	0.054
68	-0.34639	0.0049	MFNG	0.054
69	-0.39924	0.005	TGFBR2	0.054
70	-0.40818	0.005	IL1R2	0.054
71	-0.27827	0.0051	NOTCH1	0.0544
72	-0.29917	0.0053	ACVRL1	0.0551
73	-0.36763	0.0054	BNC2	0.0551
74	-0.46897	0.0055	MARCO	0.0551
75	-0.31018	0.0055	IL6R	0.0551
76	-0.75035	0.0056	DUSP4	0.0551
77	-0.14048	0.0056	SP1	0.0551
78	-0.36347	0.0059	PDE9A	0.0568
79	-0.40547	0.006	PLA2G4A	0.0571
80	-0.54627	0.0061	SOX17	0.0571
81	-0.27175	0.0062	FLI1	0.0578
82	-0.54724	0.0063	GPX3	0.0581
83	-0.55214	0.0066	CCL21	0.0593

© 2021 Robinson TJ et al. *JAMA Network Open*.

84	0.22304	0.0066	PSMB7	0.0593
85	-0.3273	0.0067	ROBO4	0.0593
86	-0.32814	0.0071	HLA_DMB	0.0616
87	-0.44584	0.0071	ENPP2	0.0616
88	-0.80713	0.0074	KRT7	0.0623
89	-0.20573	0.0074	PARP2	0.0623
90	-0.30183	0.0075	CLEC14A	0.0623
91	-0.33266	0.0077	MAML2	0.0623
92	0.45277	0.0078	UBE2C	0.0623
93	-0.50659	0.0079	CD8A	0.0623
94	-0.47941	0.0079	GDF5	0.0623
95	-0.48167	0.0079	LIF	0.0623
96	-0.38172	0.008	CD274	0.0623
97	-0.71177	0.0081	WNT11	0.0623
98	0.58424	0.0081	BIRC5	0.0623
99	-0.48839	0.0082	SOX9	0.0624
100	-0.60331	0.0086	CHIT1	0.0648
101	-0.4941	0.0088	TNN	0.0655
102	-0.30528	0.0089	SMO	0.0655
103	-0.40543	0.0091	SRPX	0.0657
104	-0.78161	0.0092	SCUBE2	0.0657
105	-0.44363	0.0093	CLEC5A	0.0657
106	-0.49266	0.0093	INHBB	0.0657
107	-0.39084	0.0093	SOCS1	0.0657
108	-0.48482	0.0094	GZMB	0.0657
109	-0.62719	0.0097	NR4A3	0.0667
110	-0.39145	0.01	MLLT3	0.0686
111	-0.42017	0.0103	PALMD	0.0698
112	-0.24644	0.0105	TLR4	0.0705
113	0.23331	0.0106	PCNA	0.0706
114	-0.36298	0.0107	TEK	0.0708
115	-0.5096	0.0111	WNT10A	0.0727
116	-0.34022	0.0113	IL10RA	0.0729
117	-0.37667	0.0114	EMCN	0.0729
118	-0.5135	0.0116	DPT	0.0729
119	-0.32507	0.0116	PDGFRA	0.0729
120	-0.46105	0.0116	CCL4	0.0729
121	-0.31872	0.012	PIK3R5	0.0747
122	-0.67196	0.0121	SERPINB5	0.0747
123	-0.32679	0.0123	HLA_C	0.0748
124	-0.31176	0.0123	DUSP6	0.0748
125	-0.39487	0.0127	NPR1	0.0764
126	-0.63836	0.0129	GABRP	0.0767

© 2021 Robinson TJ et al. *JAMA Network Open*.

127	-0.51892	0.013	WIF1	0.0768
128	-0.2501	0.0132	MAF	0.0774
129	-0.64267	0.0135	PLA2G2A	0.0782
130	-0.51821	0.0135	IKZF3	0.0782
131	-0.33638	0.0137	PRKCA	0.0786
132	-0.26537	0.0139	JAK2	0.0791
133	-0.59506	0.0142	TIMP4	0.0791
134	-0.46492	0.0142	IL7R	0.0791
135	0.39031	0.0143	KIFC1	0.0791
136	-0.45043	0.0143	GPC4	0.0791
137	-0.15	0.0144	NCAPH2	0.0791
138	-0.31432	0.0146	CD34	0.0795
139	-0.50282	0.0149	CCL5	0.0802
140	-0.51587	0.015	SFRP4	0.0802
141	-0.49937	0.0151	NKG7	0.0802
142	-1.32093	0.0154	SOX10	0.0802
143	-0.36175	0.0154	BMP6	0.0802
144	-0.28414	0.0154	DDR2	0.0802
145	-0.45813	0.0157	LAMB3	0.0802
146	-0.38586	0.0158	FGF13	0.0802
147	-0.44547	0.016	GZMA	0.0802
148	0.15755	0.0161	SKP2	0.0802
149	-0.34969	0.0161	BMP2	0.0802
150	-0.24541	0.0162	KAT2B	0.0802
151	-0.34353	0.0163	SHE	0.0802
152	-0.32973	0.0164	EDNRB	0.0802
153	-0.43929	0.0165	CXCL8	0.0802
154	-0.43042	0.0165	CD8B	0.0802
155	0.43408	0.0166	TOP2A	0.0802
156	-0.3772	0.0166	JAK3	0.0802
157	-0.55923	0.0171	LAMC2	0.082
158	-0.53286	0.0173	SELE	0.0823
159	-0.3454	0.0174	CACNA2D1	0.0823
160	-0.49152	0.0176	ID4	0.0825
161	-0.36375	0.0178	CCR1	0.0828
162	-0.37841	0.0179	ALDH1A1	0.0828
163	-0.482	0.0179	VIT	0.0828
164	0.38122	0.0191	FAM83D	0.0874
165	-0.27726	0.0193	IL4R	0.0881
166	-0.44978	0.0199	TMPRSS2	0.09
167	-0.43004	0.0204	KLRK1	0.0917
168	-0.39013	0.0205	FZD10	0.092
169	-0.36398	0.0208	TNFAIP6	0.0922

© 2021 Robinson TJ et al. *JAMA Network Open*.

170	-0.44145	0.0209	ITGB6	0.0922
171	-0.34845	0.021	FOXC1	0.0922
172	0.69382	0.0214	SIX1	0.0936
173	-0.28113	0.0215	CYBB	0.0936
174	-0.29385	0.022	LIFR	0.095
175	0.27796	0.0224	HIST1H2BH	0.0964
176	-0.44178	0.0229	KCNB1	0.0972
177	0.26557	0.023	RAC3	0.0972
178	-0.41912	0.0231	DCN	0.0972
179	-0.30798	0.0231	HGF	0.0972
180	-0.40729	0.0235	VEGFD	0.098
181	0.40819	0.024	CEP55	0.0993
182	-0.43345	0.024	LEMD1	0.0993
183	-0.3321	0.0242	ZFPM2	0.0996
184	-0.415	0.0245	PPARGC1A	0.1
185	-0.36934	0.0247	HLA_DPB1	0.1004
186	-0.54963	0.0249	SLPI	0.1004
187	-0.5077	0.025	HSPA2	0.1004
188	-0.43661	0.0251	TMEM45B	0.1004
189	-0.37118	0.0254	CCR5	0.1009
190	-0.38757	0.0261	PDCD1	0.1032
191	-0.36886	0.0262	CXCR6	0.1032
192	-0.37918	0.0276	FGL2	0.1076
193	-0.18035	0.0276	ITGAV	0.1076
194	-0.41173	0.0281	CLDN1	0.1082
195	-0.32538	0.0283	ITGA6	0.1082
196	0.35806	0.0283	TYMS	0.1082
197	-0.80849	0.0283	FGF10	0.1082
198	-0.27975	0.0288	BCL6B	0.1094
199	0.35072	0.029	CENPF	0.1096
200	-0.32484	0.0293	FGF18	0.1103
201	-0.35884	0.0297	DTX4	0.1112
202	-0.39738	0.0299	CCR2	0.1114
203	-0.41908	0.0301	PTGER3	0.1115
204	0.18061	0.0305	PIK3CA	0.1123
205	-0.34325	0.0309	RORB	0.1135
206	-0.30999	0.0318	CDKN1C	0.116
207	0.36408	0.0319	CDK1	0.116
208	-0.26528	0.0323	DLL1	0.1169
209	-0.40047	0.0326	FOXC2	0.1173
210	0.17483	0.0336	PIK3R2	0.1201
211	-0.38761	0.0338	ARNT2	0.1201
212	0.36576	0.034	ESR1	0.1201

© 2021 Robinson TJ et al. *JAMA Network Open*.

213	-0.23823	0.0342	ID2	0.1201
214	-0.37471	0.0343	ADAM12	0.1201
215	0.34522	0.0343	MKI67	0.1201
216	-0.34054	0.0352	HAS1	0.1222
217	0.34222	0.0353	HIST1H3H	0.1222
218	-0.43036	0.0355	IL22RA2	0.1226
219	-0.41074	0.0358	GADD45G	0.1228
220	-0.27951	0.0368	PDCD1LG2	0.1258
221	-0.50215	0.0371	ASPN	0.1258
222	-0.3197	0.0375	GRIN2A	0.1258
223	-0.55426	0.0375	IRX1	0.1258
224	-0.43293	0.0375	OGN	0.1258
225	0.23988	0.0378	HIST1H1C	0.1262
226	-0.30275	0.0379	TNF	0.1262
227	-0.47462	0.039	LTB	0.1291
228	-0.35177	0.0395	MET	0.1304
229	-0.24653	0.0399	HEG1	0.1309
230	0.77611	0.0404	PPP2R2C	0.1312
231	-0.37213	0.0406	RARRES3	0.1312
232	-0.45933	0.0406	PLCE1	0.1312
233	-0.32322	0.0406	FAP	0.1312
234	-0.34112	0.0419	HLA_DPA1	0.1346
235	0.16882	0.0425	BMPR1A	0.136
236	-0.29984	0.0431	RAC2	0.1372
237	0.30533	0.0434	PTTG1	0.1376
238	0.4224	0.0439	PKMYT1	0.1383
239	-0.40088	0.044	BBOX1	0.1383
240	-0.32598	0.0442	HLA_DRA	0.1386
241	-0.2527	0.0457	VIM	0.1423
242	-0.509	0.0458	PLA2G3	0.1423
243	-0.33019	0.0461	PRKCB	0.1427
244	-0.29938	0.0464	ECM2	0.1429
245	-0.1661	0.0472	PARP4	0.1436
246	-0.39066	0.0472	EFNA5	0.1436
247	-0.38696	0.0473	CCL3L1	0.1436
248	-0.44233	0.0474	WNT5A	0.1436
249	-0.22447	0.0484	HLA_DMA	0.1459
250	-0.26626	0.0486	ZEB1	0.1459
251	-0.40267	0.0488	RELN	0.1459
252	-0.19591	0.0489	SMAD3	0.1459
253	-0.38189	0.0498	TTYH1	0.148

© 2021 Robinson TJ et al. *JAMA Network Open*.

T2 vs. T1 limited to Luminal A tumors – Genes with p ≤ 0.05 shown

Obs	*Estimate*	*ProbChiSq*	*Gene*	*FDR*
1	-0.92388	0.0002	SOCS3	0.0706
2	-0.92278	0.0003	CXCL8	0.0706
3	-0.80081	0.0003	SOX9	0.0706
4	0.61725	0.0005	PBX3	0.0852
5	-0.73515	0.0008	FOSL1	0.1116
6	-0.82402	0.0009	SELE	0.1116
7	-0.55833	0.0021	ICAM1	0.1746
8	-0.75574	0.0021	GZMB	0.1746
9	-0.70354	0.0021	PTGS2	0.1746
10	-0.64701	0.0025	CCL2	0.1746
11	-0.19592	0.0032	NCAPH2	0.1746
12	-1.05219	0.0032	KRT7	0.1746
13	-0.61319	0.0032	PIM1	0.1746
14	-0.50051	0.0035	NFATC1	0.1746
15	-0.92743	0.0036	NR4A3	0.1746
16	-0.53422	0.0045	TNFAIP6	0.1746
17	-0.58287	0.0045	SNAI1	0.1746
18	-0.60182	0.0048	BCL2A1	0.1746
19	-0.61829	0.0048	INHBB	0.1746
20	-0.87846	0.005	IL6	0.1746
21	-0.65049	0.005	GPC4	0.1746
22	-0.63949	0.0054	KIT	0.1746
23	-0.71278	0.0055	TMEM45B	0.1746
24	-0.58807	0.0056	TWIST2	0.1746
25	-0.45174	0.0071	CXCL12	0.2064
26	-0.24474	0.0071	BORCS7	0.2064
27	-0.5138	0.0084	RORB	0.2285
28	-0.81887	0.0085	SERPINB5	0.2285
29	0.40246	0.0091	CCNB1	0.2301
30	-0.59156	0.0092	MARCO	0.2301
31	-0.55666	0.0097	CACNG1	0.2347
32	-0.58689	0.0112	TMPRSS2	0.2641
33	-0.46916	0.0126	PLA2G4A	0.2712
34	-0.70765	0.0126	HSPA2	0.2712
35	-0.56931	0.0134	PPARGC1A	0.2712
36	-0.63463	0.0139	SOX17	0.2712
37	-0.45456	0.0139	SYTL4	0.2712
38	-0.44563	0.0142	CD274	0.2712
39	-0.35289	0.0146	SMO	0.2712
40	-0.38857	0.0148	SPRY2	0.2712

© 2021 Robinson TJ et al. *JAMA Network Open*.

41	-0.56854	0.0148	LIF	0.2712
42	-0.21923	0.0153	EIF4E2	0.2712
43	-0.54521	0.0159	LAMB3	0.2712
44	-0.5159	0.0163	PDCD1	0.2712
45	0.30529	0.0167	HDAC2	0.2712
46	-0.39568	0.0169	PDE9A	0.2712
47	-0.24049	0.017	PARP2	0.2712
48	-0.7818	0.0175	WNT11	0.2712
49	-0.74101	0.0179	SLPI	0.2712
50	-0.34412	0.018	IL4R	0.2712
51	-0.48445	0.0185	SOCS1	0.2722
52	-0.48064	0.0191	HAS1	0.2766
53	-0.63191	0.0206	CACNA2D3	0.2909
54	-0.24916	0.0209	DDX39A	0.2909
55	-0.52533	0.0214	IL1RN	0.2925
56	-0.5141	0.0219	CCR1	0.2942
57	-0.3393	0.0252	ID2	0.3324
58	-0.7231	0.0263	KRT17	0.3324
59	-0.58499	0.0267	CCNA1	0.3324
60	-0.44901	0.0277	LEPR	0.3324
61	-0.5046	0.0278	TNN	0.3324
62	-0.43673	0.0279	EGFR	0.3324
63	-0.57117	0.0285	LAD1	0.3324
64	-0.5079	0.0287	DKK1	0.3324
65	-0.60124	0.0291	IL22RA2	0.3324
66	-0.51483	0.0292	PTGER3	0.3324
67	-0.83724	0.0308	CHAD	0.3447
68	-0.48802	0.0312	CLEC5A	0.3447
69	-0.42656	0.0325	DTX1	0.3509
70	-0.38309	0.0331	FOXC1	0.3509
71	0.28737	0.0331	CDKN1B	0.3509
72	-0.48263	0.0337	GDF5	0.3521
73	-0.63647	0.0362	GABRP	0.3725
74	0.17318	0.0374	SKP2	0.3805
75	0.25383	0.0388	PTEN	0.3862
76	-0.44387	0.039	PPARG	0.3862
77	-0.58527	0.0395	WNT5A	0.3862
78	-0.5371	0.0409	LEMD1	0.3929
79	-0.51071	0.0413	BBOX1	0.3929
80	-0.41605	0.0419	PLCB4	0.3936
81	0.21902	0.044	SKP1	0.4074
82	0.69943	0.0449	DNAJC12	0.4074
83	-0.30025	0.0457	MYCT1	0.4074

© 2021 Robinson TJ et al. *JAMA Network Open*.

84	-0.63464	0.0464	NR4A1	0.4074
85	-0.52554	0.0466	GADD45G	0.4074
86	-0.41496	0.0466	TNF	0.4074
87	-0.50466	0.0473	FHL1	0.4085
88	-0.34918	0.0483	FZD9	0.4124

© 2021 Robinson TJ et al. *JAMA Network Open*.

T2 vs. T1 limited to Luminal B tumors – Genes with p ≤ 0.05 shown

Obs	*Estimate*	*ProbChiSq*	*Gene*	*FDR*
1	-0.95083	0.0005	HLA_DPA1	0.1324
2	-0.65546	0.0007	CYBB	0.1324
3	-0.89046	0.0008	HLA_DPB1	0.1324
4	-0.56965	0.0012	FLI1	0.1324
5	-0.58995	0.0013	KAT2B	0.1324
6	-0.71312	0.0016	RORA	0.1324
7	-1.01917	0.0018	GPX3	0.1324
8	-0.52235	0.0019	ELK3	0.1324
9	0.7043	0.0022	RAC3	0.1324
10	-0.70144	0.0024	HLA_DRA	0.1324
11	-0.52006	0.0026	PECAM1	0.1324
12	-0.58599	0.0027	CD84	0.1324
13	-0.5996	0.0028	PIK3CG	0.1324
14	-0.66377	0.0029	RUNX3	0.1324
15	-0.54271	0.003	HLA_DMB	0.1324
16	-0.52639	0.0032	ZEB2	0.1324
17	-0.62834	0.0034	CD68	0.1324
18	-0.83612	0.0034	GZMH	0.1324
19	-0.58239	0.0036	CCND2	0.1324
20	-0.53026	0.0037	HLA_E	0.1324
21	2.43033	0.0037	DHRS2	0.1324
22	-1.12471	0.0046	CACNA1D	0.1586
23	-0.73102	0.0055	FGL2	0.1814
24	-2.09267	0.0058	CEACAM5	0.1831
25	-0.56171	0.0075	PIK3R5	0.2232
26	-0.48895	0.0081	PSMB10	0.2232
27	-0.39279	0.0082	SMAD5	0.2232
28	-0.43917	0.0089	TLR4	0.2232
29	-0.4195	0.0091	ATM	0.2232
30	-0.55892	0.0091	TGFBR2	0.2232
31	-0.78703	0.0092	LPL	0.2232
32	-0.72116	0.0106	CCR2	0.2305
33	-1.27924	0.0106	CXCL9	0.2305
34	-0.60568	0.0108	RAC2	0.2305
35	-0.95365	0.0112	LEP	0.2305
36	-0.72227	0.0112	CCL3L1	0.2305
37	1.22061	0.0114	SIX1	0.2305
38	-1.25593	0.0117	DUSP4	0.2305
39	-0.42991	0.012	HLA_DMA	0.2305
40	1.22563	0.0127	AREG	0.2332

© 2021 Robinson TJ et al. *JAMA Network Open*.

41	-0.51381	0.0127	PIK3R1	0.2332
42	-0.8067	0.013	CD8A	0.2334
43	-0.65148	0.0141	CCR5	0.2431
44	0.9666	0.0145	EREG	0.2431
45	-0.85347	0.0148	CCL5	0.2431
46	-0.40943	0.0149	CD34	0.2431
47	-0.48316	0.0157	PRKCA	0.2494
48	0.55698	0.0161	HES1	0.2494
49	-0.75381	0.0165	GZMA	0.2494
50	-0.83981	0.0166	NKG7	0.2494
51	-0.38138	0.0169	MAF	0.2497
52	0.25544	0.0186	PALB2	0.2634
53	-0.70357	0.0186	HLA_B	0.2634
54	-0.56569	0.0194	HLA_C	0.2704
55	-0.58043	0.0201	CBLC	0.275
56	0.54461	0.0212	ALDOA	0.2846
57	-0.81171	0.022	IL2RB	0.2901
58	0.84863	0.0233	SOCS2	0.2943
59	-0.68583	0.0236	CCL4	0.2943
60	-0.37947	0.0242	ACVRL1	0.2943
61	0.47565	0.0244	UBE2C	0.2943
62	0.46548	0.0247	FAM83D	0.2943
63	-0.49821	0.025	SPN	0.2943
64	-0.50173	0.0252	MFNG	0.2943
65	-0.47623	0.0254	PIK3CD	0.2943
66	-0.45819	0.026	CAV1	0.2947
67	0.36935	0.0266	SLC2A1	0.2947
68	-0.77279	0.0266	F3	0.2947
69	0.48818	0.0288	TYMS	0.3139
70	-0.46497	0.0293	IL10RA	0.315
71	-0.93275	0.0305	HOXB3	0.3184
72	-0.58278	0.0305	ITGA6	0.3184
73	0.71083	0.0309	BIRC5	0.3184
74	1.20579	0.0319	BAMBI	0.3237
75	0.4602	0.0343	E2F5	0.3417
76	-0.35608	0.0356	MSR1	0.3417
77	-0.84039	0.0356	PLA2G2A	0.3417
78	-0.61307	0.0358	CRYAB	0.3417
79	0.4872	0.036	CEP55	0.3417
80	0.30895	0.0364	MAD2L1	0.3417
81	-0.77988	0.0373	TIGIT	0.3465
82	0.49124	0.0386	BNIP3	0.3524
83	-0.41096	0.0395	CDC14B	0.3524

© 2021 Robinson TJ et al. *JAMA Network Open*.

84	-0.79515	0.0398	THBS4	0.3524
85	-0.50847	0.0398	ENPP2	0.3524
86	-0.3216	0.0412	RBL2	0.3578
87	-0.74399	0.0417	COL27A1	0.3578
88	-0.68859	0.0421	RARRES3	0.3578
89	-0.32187	0.0423	PTEN	0.3578
90	-0.50152	0.0434	DTX1	0.3626
91	0.41044	0.0439	AXIN2	0.3627
92	-1.26982	0.0451	AGTR1	0.3643
93	-0.61532	0.046	PSMB9	0.3643
94	0.37399	0.0463	HIST1H2BH	0.3643
95	0.86764	0.0463	CACNG4	0.3643
96	-0.57634	0.0473	FHL1	0.3643
97	-0.66755	0.0476	WNT10A	0.3643
98	0.85601	0.0494	WT1	0.3643
99	0.33127	0.0496	CDC20	0.3643
100	-0.40392	0.0498	CMKLR1	0.3643
1	-0.95083	0.0005	HLA_DPA1	0.1324
2	-0.65546	0.0007	CYBB	0.1324
3	-0.89046	0.0008	HLA_DPB1	0.1324
4	-0.56965	0.0012	FLI1	0.1324
5	-0.58995	0.0013	KAT2B	0.1324
6	-0.71312	0.0016	RORA	0.1324
7	-1.01917	0.0018	GPX3	0.1324
8	-0.52235	0.0019	ELK3	0.1324
9	0.7043	0.0022	RAC3	0.1324
10	-0.70144	0.0024	HLA_DRA	0.1324
11	-0.52006	0.0026	PECAM1	0.1324
12	-0.58599	0.0027	CD84	0.1324

© 2021 Robinson TJ et al. *JAMA Network Open*.

List of all gene symbols (N = 752) analyzed using the Nanostring BC360 panel.

ABCA8	BCAS1	CCL7	CDKN1A	CXADR	EIF2AK3	FHL1	GREM1	HLA_E
ACTR3B	BCL11A	CCL8	CDKN1B	CXCL10	EIF3B	FLI1	GRIA3	HMGA1
ACVR1B	BCL2	CCNA1	CDKN1C	CXCL12	EIF4E2	FLNC	GRIN1	HNF1A
ACVR1C	BCL2A1	CCNA2	CDKN2A	CXCL13	EIF4EBP1	FLRT3	GRIN2A	HOXA5
ACVRL1	BCL2L1	CCNB1	CDKN2B	CXCL5	ELF3	FLT3	GSK3B	HOXA7
ADAM12	BCL6B	CCND1	CDKN2C	CXCL8	ELK3	FNBP1	GTF2H2	HOXA9
ADCY9	BDNF	CCND2	CDKN2D	CXCL9	ELOVL2	FOS	GZMA	HOXB13
ADD1	BIRC5	CCNE1	CDKN3	CXCR6	EMCN	FOSL1	GZMB	HOXB3
ADM	BLM	CCNE2	CEACAM5	CXorf36	ENO1	FOXA1	GZMH	HSPA2
AGR2	BLVRA	CCR1	CEACAM6	CXXC5	ENPP2	FOXC1	GZMM	IBSP
AGT	BMP2	CCR2	CENPF	CYBB	EP300	FOXC2	HAPLN1	ICAM1
AGTR1	BMP4	CCR5	CEP55	CYP4F3	EPAS1	FOXM1	HAS1	ID1
AKT3	BMP5	CD163	CFD	DCN	ERBB2	FOXP3	HBB	ID2
ALDH1A1	BMP6	CD19	CHAD	DDB2	ERBB4	FREM2	HDAC1	ID4
ALDOA	BMP7	CD1E	CHEK2	DDR2	EREG	FST	HDAC10	IDO1
ANGPT1	BMP8A	CD24	CHI3L1	DDX39A	ESPL1	FSTL1	HDAC11	IFT140
ANLN	BMPR1A	CD27	CHIT1	DEPDC1	ESR1	FSTL3	HDAC2	IGF1
ANXA9	BMPR1B	CD274	CHRNA5	DHRS2	ETV4	FUT3	HDAC5	IGF1R
APH1B	BMPR2	CD276	CKB	DKK1	ETV7	FXYD3	HDAC6	IKZF3
APOD	BNC2	CD34	CKMT1A	DKK2	EXO1	FZD10	HDC	IL10RA
APOE	BNIP3	CD36	CKS1B	DLGAP5	EYA1	FZD7	HEG1	IL11RA
AR	BORCS7	CD44	CLDN1	DLL1	EYA2	FZD8	HELLS	IL12RB2
AREG	BRCA1	CD68	CLDN3	DLL3	EYA4	FZD9	HEMK1	IL13RA1
ARID1A	BRCA2	CD84	CLDN4	DLL4	F3	GABRP	HES1	IL1B
ARNT2	BTG2	CD8A	CLDN7	DNAJC12	FAM124B	GADD45A	HGF	IL1R2
ASPM	C5orf38	CD8B	CLEC14A	DPT	FAM198B	GADD45B	HIF1A	IL1RN
ASPN	CA12	CDC14A	CLEC5A	DSC2	FAM214A	GADD45G	HIST1H1C	IL20RA
ATAD2	CACNA1D	CDC14B	CMKLR1	DTX1	FAM83D	GAS1	HIST1H2BH	IL20RB
ATM	CACNA1H	CDC20	CNTFR	DTX3	FANCF	GATA3	HIST1H3H	IL22RA2
ATP10B	CACNA2D1	CDC25A	COL11A1	DTX4	FAP	GATA4	HIST3H2BB	IL24
AURKA	CACNA2D3	CDC25B	COL27A1	DUSP4	FBN1	GDF15	HK2	IL2RA
AURKB	CACNG1	CDC25C	COL2A1	DUSP6	FGF1	GDF5	HLA_A	IL2RB
AXIN1	CACNG4	CDC6	COL4A6	E2F1	FGF10	GGH	HLA_B	IL3RA
AXIN2	CACNG6	CDC7	COL6A3	E2F5	FGF12	GHR	HLA_C	IL4R
B3GNT3	CALML5	CDCA5	COL7A1	ECM2	FGF13	GJB2	HLA_DMA	IL6
BAD	CAMK2B	CDCA7L	COL9A3	EDN1	FGF18	GLI3	HLA_DMB	IL6R
BAG1	CAV1	CDCA8	COLEC12	EDNRB	FGF2	GNG4	HLA_DOB	IL7R
BAIAP2L1	CBLC	CDH1	COMP	EFNA3	FGF7	GNLY	HLA_DPA1	INHBA
BAIAP3	CCL2	CDH2	CPA3	EFNA5	FGF9	GPC4	HLA_DPB1	INHBB
BAMBI	CCL21	CDH3	CREBBP	EGF	FGFR2	GPR160	HLA_DQA1	IRF6
BAX	CCL3L1	CDH5	CRYAB	EGFR	FGFR3	GPX3	HLA_DQB1	IRX1

© 2021 Robinson TJ et al. *JAMA Network Open*.

BBC3	CCL4	CDK1	CSF3R	EGLN2	FGFR4	GRB2	HLA_DRA	ISG15
BBOX1	CCL5	CDK6	CTSW	EGLN3	FGL2	GRB7	HLA_DRB1	ISM1
ITGA6	LINC02381	MUC1	PALB2	POPDC3	RBX1	SMAD3	TGFB3	WEE1
ITGAV	LPL	MUS81	PALMD	PPARG	RELN	SMAD4	TGFBR2	WIF1
ITGB1	LRP2	MYBL2	PARP1	PPARGC1A	RFC4	SMAD5	THBS1	WNT10A
ITGB3	LRRC32	MYC	PARP2	PPP2CB	RNASE2	SMC1B	THBS2	WNT11
ITGB6	LTB	MYCN	PARP4	PPP2R1A	RNF103	SMO	THBS4	WNT2
ITPR1	LTBP1	MYCT1	PAX5	PPP2R2C	ROBO4	SMURF2	THY1	WNT4
JAG1	MAD2L1	NASP	PAX8	PRC1	ROCK1	SNAI1	TIE1	WNT5A
JAG2	MAF	NAT1	PBX3	PREP	ROCK2	SNAI2	TIGIT	WNT5B
JAK1	MAML2	NCAM1	PCK1	PRF1	RORA	SOCS1	TIMP4	WNT6
JAK2	MAP2K4	NCAPH2	PCNA	PRKAA2	RORB	SOCS2	TLE3	WNT7B
JAK3	MAP3K12	NDC80	PDCD1	PRKACA	RPS6KA5	SOCS3	TLR4	WRN
JAM2	MAPK1	NDP	PDCD1LG2	PRKACB	RPS6KB1	SOX10	TLX1	WT1
JCAD	MAPK10	NEIL1	PDE9A	PRKCA	RPS6KB2	SOX17	TMEM45B	XRCC2
JUN	MAPK3	NEIL3	PDGFB	PRKCB	RRM2	SOX2	TMPRSS2	XRCC3
KAT2B	MAPK8IP2	NEO1	PDGFRA	PRKDC	RUNX3	SOX9	TMPRSS4	ZBTB16
KCNB1	MAPT	NETO2	PDGFRB	PRKX	S100A14	SP1	TNF	ZEB1
KDR	MARCO	NFATC1	PDK4	PRLR	S100A7	SPC25	TNFAIP6	ZEB2
KIAA0040	MCM2	NFKBIZ	PECAM1	PROM1	S1PR1	SPDEF	TNFSF10	ZFPM2
KIF11	MCM3	NGFR	PFDN2	PSAT1	SCARA5	SPN	TNKS	ZFYVE9
KIF14	MDM2	NKG7	PGK1	PSMB10	SCUBE2	SPP1	TNKS2	ZIC2
KIF23	MED1	NOD2	PGR	PSMB7	SELE	SPRY1	TNN	ZNF205
KIF2C	MELK	NOTCH1	PHGDH	PSMB9	SERBP1	SPRY2	TOP2A	
KIFC1	MEOX2	NOTCH2	PIK3CA	PTCH1	SERPINB5	SPRY4	TP53	
KIT	MET	NOTCH3	PIK3CD	PTEN	SERPINH1	SRPX	TPSAB1	
KLRK1	MFNG	NPEPPS	PIK3CG	PTGDS	SFN	ST6GALNAC2	TRIP13	
KRT14	MIA	NPR1	PIK3R1	PTGER3	SFRP1	STAT1	TSPAN1	
KRT17	MIS18A	NR4A1	PIK3R2	PTGS2	SFRP2	STC1	TSPAN7	
KRT5	MKI67	NR4A3	PIK3R3	PTTG1	SFRP4	SUV39H2	TTK	
KRT6B	MLH1	NRCAM	PIK3R5	PYCARD	SHC2	SYTL4	TTYH1	
KRT7	MLLT3	NRXN1	PIM1	RAC2	SHC4	TAP1	TUBA4A	
LAD1	MLPH	NRXN3	PIP	RAC3	SHE	TAP2	TWIST1	
LAG3	MME	NSD1	PKMYT1	RAD51	SHMT2	TAPBP	TWIST2	
LAMA3	MMP11	NSD3	PLA2G2A	RAD51C	SIDT1	TBC1D9	TYK2	
LAMB3	MMP14	NTRK2	PLA2G3	RAD52	SIGIRR	TBX1	TYMP	
LAMC2	MMP3	NUDT1	PLA2G4A	RAD54L	SIX1	TCEAL1	TYMS	
LEF1	MMP7	NUF2	PLA2G4F	RARRES3	SKA3	TCF4	UBE2C	
LEFTY2	MMP9	NUMBL	PLAT	RASAL1	SKP1	TCF7L1	UBE2T	
LEMD1	MMRN2	NUPR1	PLCB1	RASGRF1	SKP2	TEK	VCAN	
LEP	MRE11	OAS3	PLCB4	RASGRF2	SLC2A1	TFDP1	VEGFA	
LEPR	MS4A2	OCLN	PLCE1	RASGRP1	SLC39A6	TFF1	VEGFD	
LFNG	MSR1	OGN	PLD1	RB1	SLC44A4	TFF3	VIM	

© 2021 Robinson TJ et al. *JAMA Network Open*.

LIF	MT1G	OLFML2B	POLD1	RBL1	SLPI	TGFB1	VIT	
LIFR	MTOR	ORC6	POLQ	RBL2	SMAD1	TGFB2	WDR77	

© 2021 Robinson TJ et al. *JAMA Network Open*.
